# Supplementary material for: YTHDF3 recognizes DNA N6-methyladenine and recruits ALKBH1 for 6mA removal from genomic DNA
Source: EMBO J. 2025 Jul 25;44(17):4899–917. doi: 10.1038/s44318-025-00512-2 (PMC12402098; doi:10.1038/s44318-025-00512-2)
Supplement: Supplementary file 1 — Appendix [file 44318_2025_512_MOESM1_ESM.pdf]

Appendix

YTHDF3 recognizes DNA N6-methyladenine and recruits ALKBH1 for 6mA removal from genomic DNA

Xin-Hui Chen[1,*], Zi-Lu Wang[1,*], Jincui Yang[1,*], Min Chen[1,*], Si-Yi Zhao[1], Kun-Xiong Guo[1], Xuelong Zheng[1], Zhengwei Zhao[1], Xiaoqiang Chen[1], Jing Li[1], Min-Min Zhang[1], Ling Ran[1], Huifang Zhu[1], Xiao-Feng Gu[2,#], Guang-Rong Yan[1,#]

[1]Biomedicine Research Center, Guangdong Provincial Key Laboratory of Major Obstetric Disease, Guangdong Provincial Clinical Research Center for Obstetrics and Gynecology, The Third Affiliated Hospital, Guangzhou Medical University, Guangzhou, 510150, China

[2]Biotechnology Research Institute, Chinese Academy of Agricultural Sciences, Beijing 100081, China

[*]These authors contributed equally to this work.

[#]Corresponding author: Prof. Guang-Rong Yan, E-mail: tgryan@jnu.edu.cn; or Prof. Xiao-Feng Gu, E-mail: guxiaofeng@caas.cn.

Table of contents

Appendix Figure S1. YTHDF3 decreases the level of 6mA in cellular genomic DNA……………………………………………………………….……..…3

Appendix Figure S2. YTHDF3 promotes the demethylation of ALKBH1 to 6mA in genomic DNA, including dsDNA……………………………………………...…4

Appendix Figure S3. YTHDF3 directly increases the demethylase activity of ALKBH1 toward 6mA in ssDNA……………………………………………...…5

Appendix Figure S4. Distribution of the 6mA-DNA peaks mediated by ALKBH1 and YTHDF3 on chromosomes and across the genome…………………………...….6

Appendix Figure S5. The DSBH domain of ALKBH1 interacts with YTHDF3…….7

Appendix Figure S6. YTHDF3 enters the cellular nucleus and recognizes and binds to 6mA in genomic DNA…………………………………………………...…8

Appendix Figure S7. N6AMT1-binding DNA fragments overlap with YTHDF3-binding DNA fragments and DNA fragments containing 6mA modifications, and N6AMT1 silencing decreases the binding of YTHDF3 to some YTHDF3-binding DNA fragments…………………………………………………….……………9

Appendix Figure S8. YTHDF3 recognizes 6mA in dsDNA and binds to ALKBH1 to recruit ALKBH1 to sites near 6mA modifications in genomic DNA, thereby facilitating the ALKBH1-mediated removal of 6mA in genomic DNA, including dsDNA…………………………………………………….…………………10

Appendix Table S1. The 6mA and A-DNA oligonucleotides used in this study….....11

Appendix Table S2. The siRNA sequences used in this study………………………15

Appendix Table S3. The RT-PCR and qPCR primers used in this study…………....16

Appendix Table S4. The biotin-labeled DNA oligonucleotides containing A or 6mA used in this study…………………………………………………………..……19

Appendix Figures

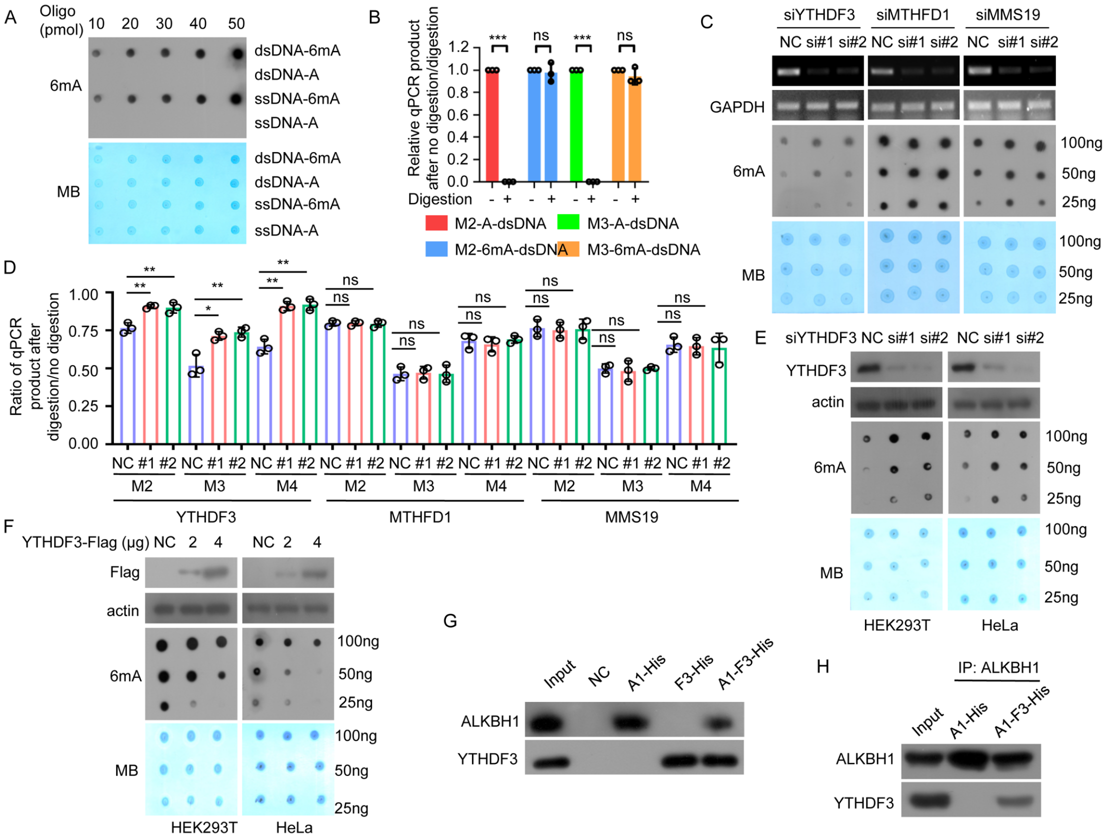

Appendix Figure S1. YTHDF3 decreases the level of 6mA in cellular genomic DNA. (A) The specificity and sensitivity of the anti-6mA antibody used in this study were detected in 6mA-modified and unmodified dsDNA or ssDNA. (B) The 130 bp synthesized M2 and M3 dsDNA oligos in which GATC was 6mA-modified or unmodified, as a standard, were validated by RE-6mA-qPCR. (C, D) Two independent siRNAs targeting the indicated genes were transfected into HCT-116 cells, and cellular genomic DNA 6mA levels were measured via dot blotting (C) and RE-6mA-Qpcr (D). (E) The cellular genomic DNA 6mA levels in Figure 1E were determined by dot blotting. (F) The cellular genomic DNA 6mA levels in Figure 1F were determined by dot blotting. (G) ALKBH1 (A1) and YTHDF3 (F3) were coexpressed in bacteria, and the ALKBH1/YTHDF3 (A1-F3) complex was copurified. ALKBH1 and YTHDF3 were detected in the recombinant ALKBH1, YTHDF3 and ALKBH1/YTHDF3 complexes. (H) The interaction of ALKBH1 with YTHDF3 was validated in the copurified recombinant ALKBH1/YTHDF3 complex. Data information: In (B, D), data are presented as mean ± SD. n=3 independent biological replicates, *p<0.05, **p<0.01, ***p<0.001, ns, non-significant (Student's t-test).

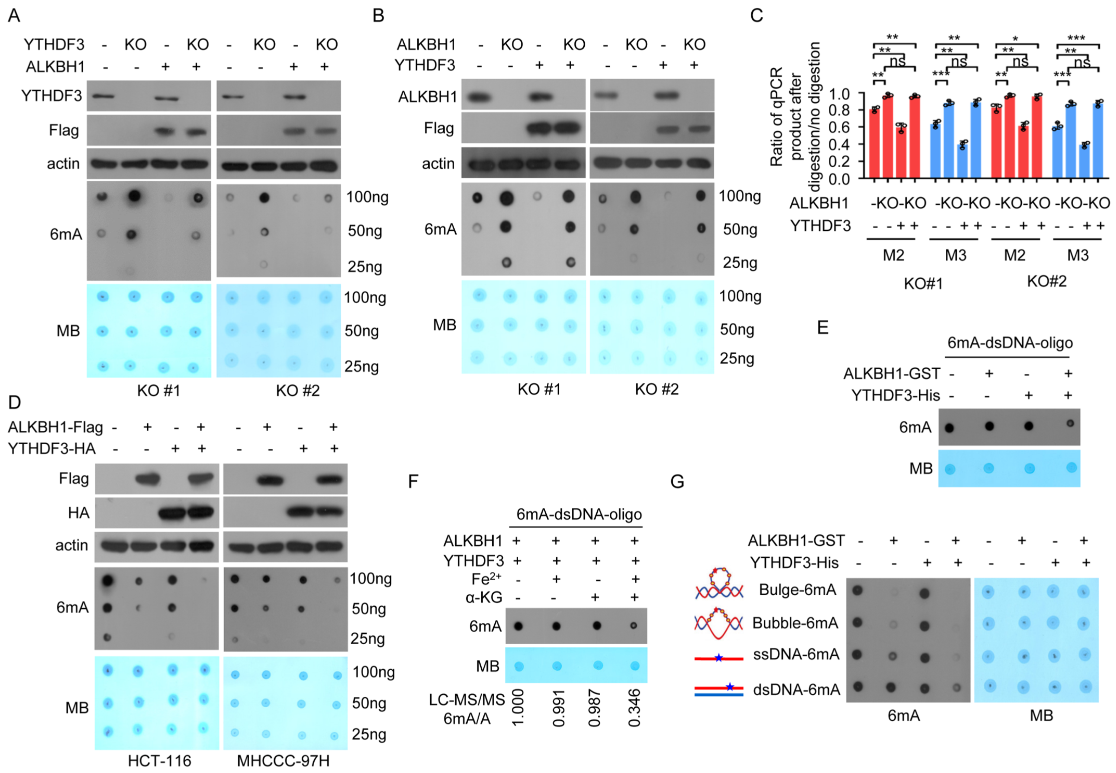

Appendix Figure S2. YTHDF3 promotes the demethylation of ALKBH1 to 6mA in genomic DNA, including dsDNA. (A) The cellular genomic DNA 6mA levels in Figure 2A were also determined via dot blotting. (B, C) YTHDF3 plasmids were transfected into ALKBH1-expressing or ALKBH1 KO HeLa cells, and cellular genomic DNA 6mA levels were determined by dot blotting (B) and RE-6mA-qPCR (C). (D) The cellular genomic DNA 6mA levels in Figure 2B were also determined via dot blotting. (E) The 6mA levels shown in Figure 2E were also determined via dot blotting. (F) The YTHDF3-mediated facilitation of the demethylase activity of ALKBH1 toward 6mA in dsDNA was dependent on Fe^{2+} and α-KG in the *in vitro* demethylation reaction. (G) The 6mA levels shown in Figure 2F were also determined via dot blotting. Data information: In (C), data are presented as mean ± SD. n=3 independent biological replicates, *p<0.05, **p<0.01, ***p<0.001, ns, non-significant (Student's t-test).

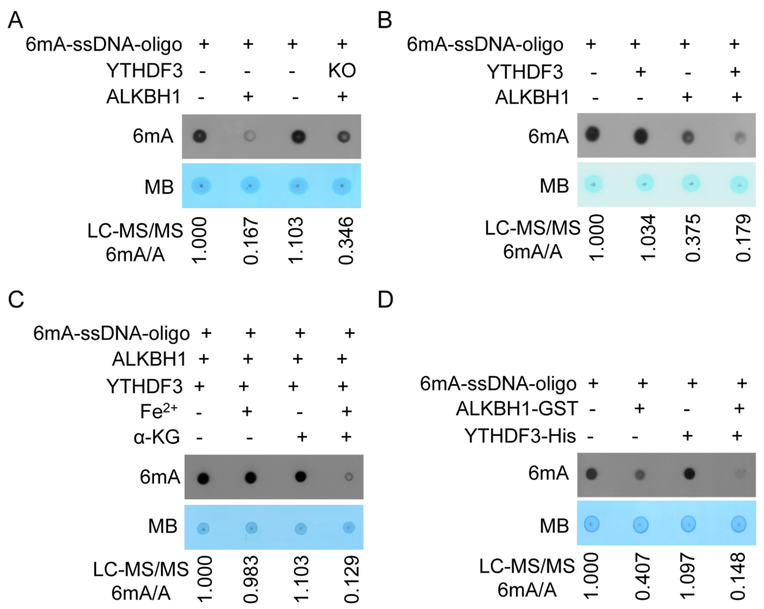

Appendix Figure S3. YTHDF3 directly increases the demethylase activity of ALKBH1 toward 6mA in ssDNA. (A) ALKBH1 immunopurified from YTHDF3-expressing and YTHDF3 KO HeLa cells was incubated with 6mA-ssDNA oligos, and the 6mA levels were determined by dot blotting and LC–MS/MS. (B) ALKBH1 immunopurified from YTHDF3 KO HeLa cells and/or YTHDF3 immunopurified from ALKBH1 KO HeLa cells was incubated with 6mA-ssDNA oligos, and the 6mA levels were determined. (C) YTHDF3-mediated facilitation of the demethylase activity of ALKBH1 toward 6mA in ssDNA was dependent on Fe^{2+} and α-KG in the *in vitro* demethylation reaction. (D) Recombinant ALKBH1 and/or recombinant YTHDF3 were incubated with 6mA-ssDNA oligos, and the 6mA levels were determined.

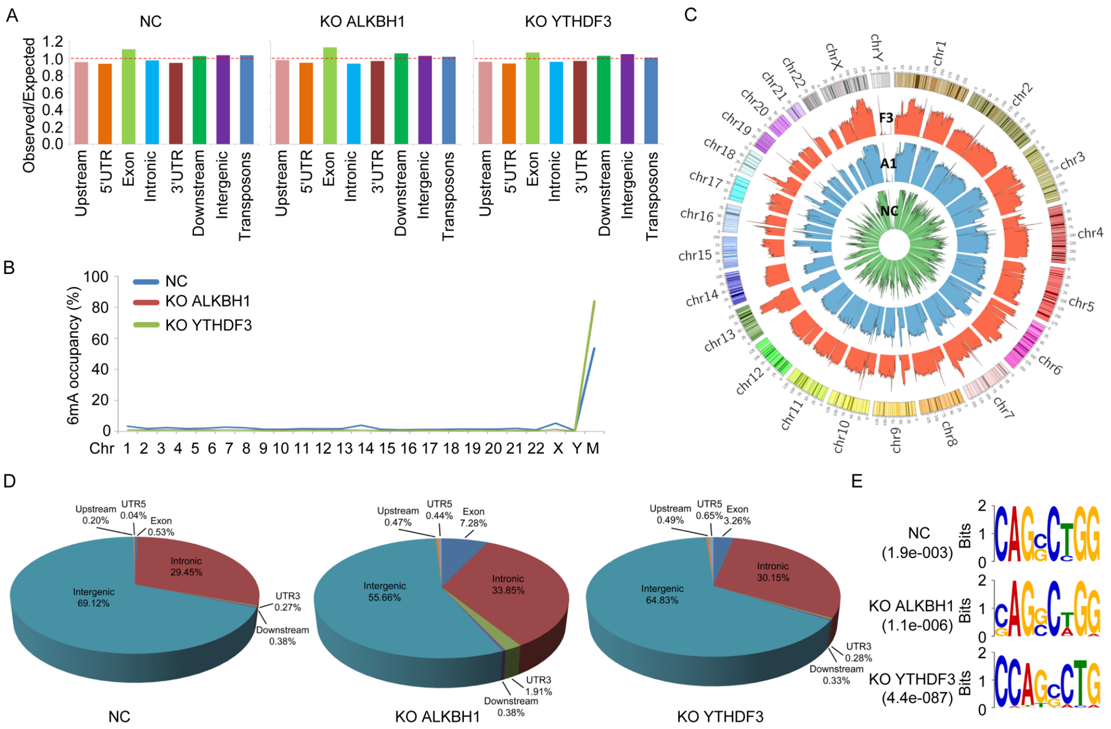

Appendix Figure S4. Distribution of the 6mA-DNA peaks mediated by ALKBH1 and YTHDF3 on chromosomes and across the genome. (A) Comparison of the observed and expected distributions of the 6mA-DNA peaks in each functional element of human genomic DNA in cells with ALKBH1 or YTHDF3 KO. (B) The 6mA-DNA peaks across all chromosomes. (C) Circos plots of the 6mA-DNA peaks across all human chromosomes in NC, ALKBH1 KO (A1) and YTHDF3 KO (F3) HeLa cells. (D) The percentage of 6mA-DNA peaks in the functional elements of human genomic DNA. (E) The common motif in the 6mA-DNA fragment peaks in the NC, ALKBH1 KO and YTHDF3 KO HeLa cells.

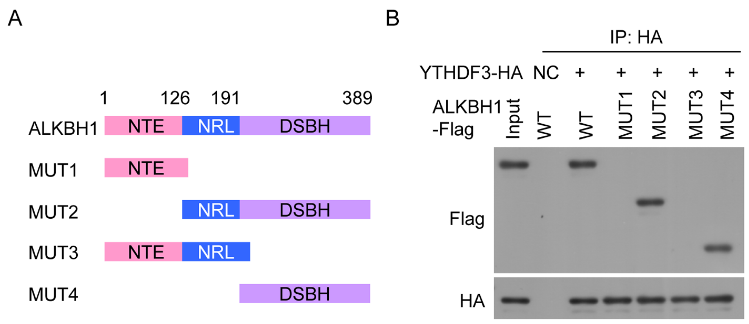

Appendix Figure S5. The DSBH domain of ALKBH1 interacts with YTHDF3. (A) Diagram of wild-type ALKBH1 and its mutant constructs with different domains. (B) The interactions of YTHDF3 with the indicated ALKBH1 mutants were detected.

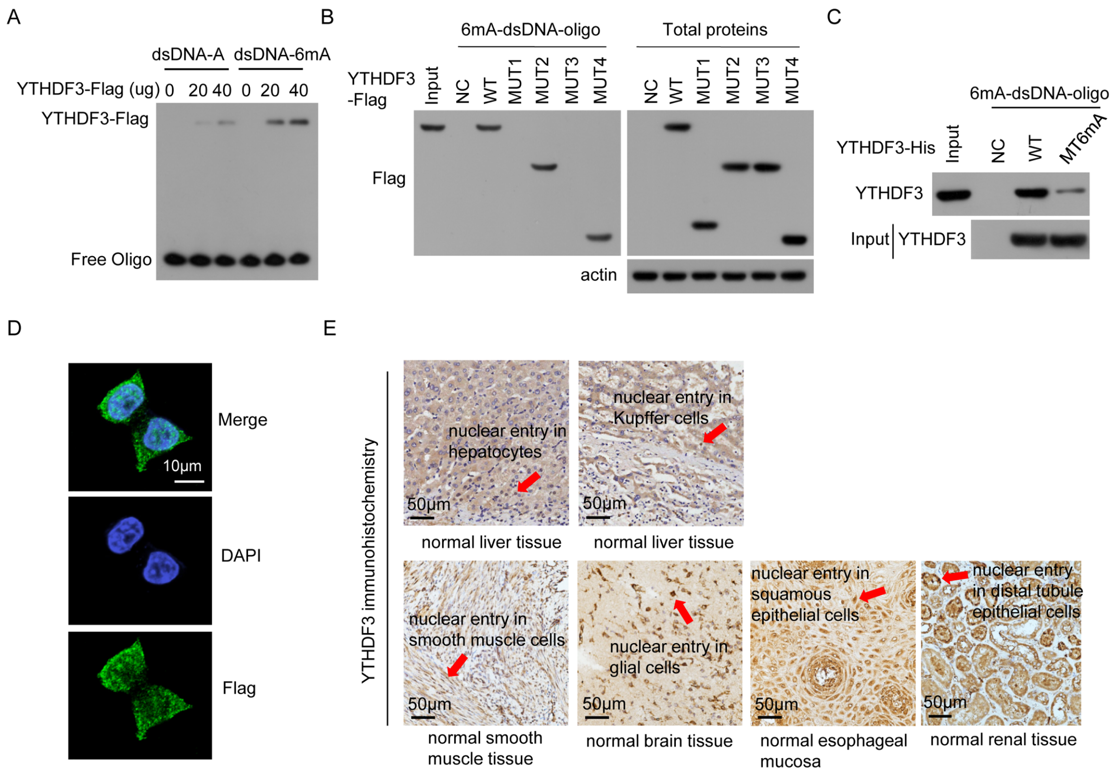

Appendix Figure S6. YTHDF3 enters the cellular nucleus and recognizes and binds to 6mA in genomic DNA. (A) The binding of YTHDF3 immunopurified from ALKBH1 KO HeLa cells to 6mA-modified and unmodified dsDNA oligos was detected by EMSA. (B) The indicated YTHDF3 mutants were transfected into HeLa cells, and the binding of the cellular YTHDF3 mutants to the 6mA-dsDNA oligos was detected. (C) Recombinant wild-type YTHDF3 and the YTHDF3 mutant MT6mA were incubated with 6mA-dsDNA oligos, and the binding of YTHDF3 to 6mA-dsDNA was evaluated. (D) YTHDF3-Flag plasmids were transfected into YTHDF3 KO HeLa cells, and YTHDF3 immunofluorescence was detected via an anti-Flag antibody. (E) The sublocalization of YTHDF3 in the indicated tissues was analyzed by IHC using an anti-YTHDF3 antibody.

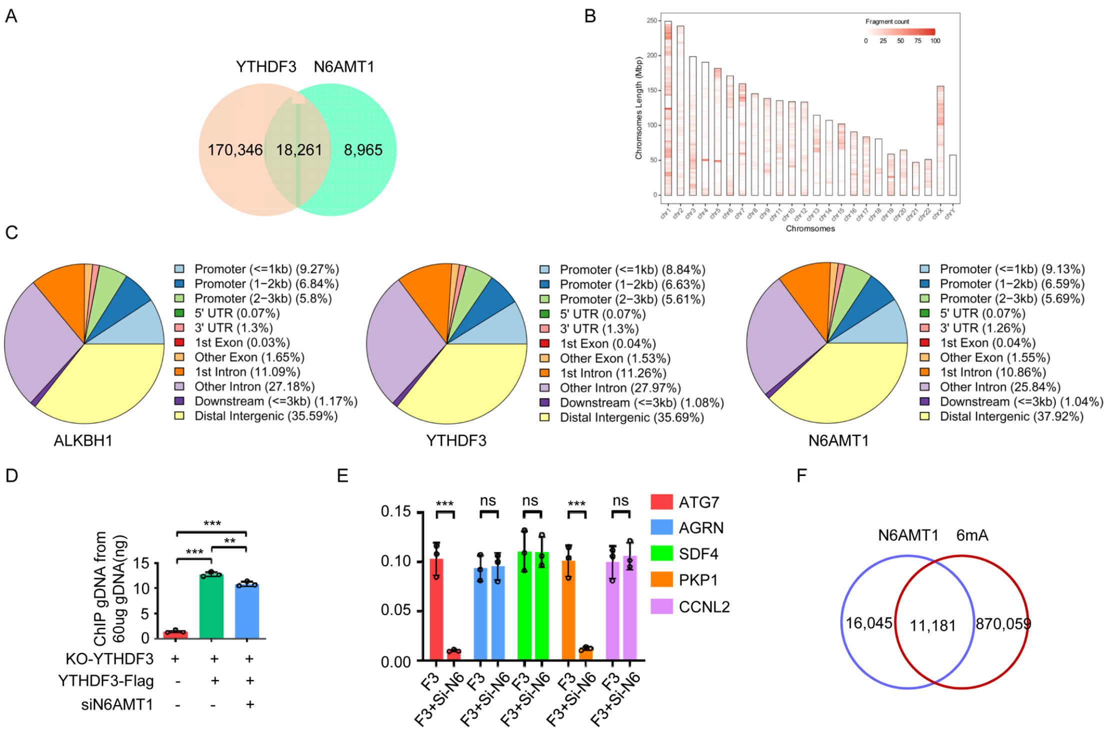

Appendix Figure S7. N6AMT1-binding DNA fragments overlap with YTHDF3-binding DNA fragments and DNA fragments containing 6mA modifications, and N6AMT1 silencing decreases the binding of YTHDF3 to some YTHDF3-binding DNA fragments. (A) Overlapping between YTHDF3 and N6AMT1 ChIP peaks. (B) N6AMT1 ChIP peaks across all chromosomes. (C) The percentages of ALKBH1, YTHDF3 and N6AMT1 ChIP peaks in the functional elements of human genomic DNA. (D) Comparison of the amount of YTHDF3 ChIP DNA between N6AMT1-expressing and N6AMT1-silenced cells. (F) Comparison of the relative amount of ChIP DNA from five randomly selected YTHDF3-binding DNA fragments containing 6mA modifications between N6AMT1-expressing and N6AMT1-silenced samples. (F) Overlapping between N6AMT1 ChIP DNA fragments and genomic DNA fragments containing the 6mA modification.

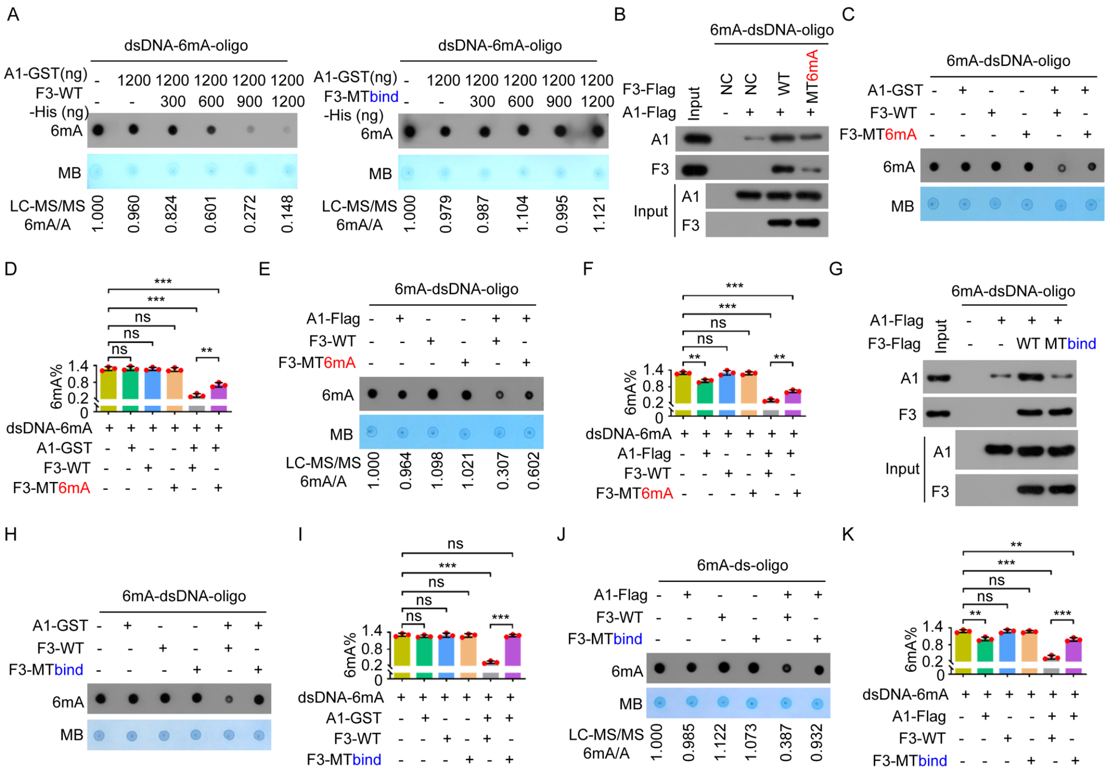

Appendix Figure S8. YTHDF3 recognizes 6mA in dsDNA and binds to ALKBH1 to recruit ALKBH1 to sites near 6mA modifications in genomic DNA, thereby facilitating the ALKBH1-mediated removal of 6mA in genomic DNA, including dsDNA. (A) The indicated amounts of recombinant ALKBH1 (A1) and recombinant YTHDF3 (F3) or its mutant MTbind were incubated with 6mA-dsDNA oligos, and the 6mA levels were determined by dot botting and LC–MS/MS. (B) ALKBH1 immunopurified from YTHDF3 KO cells and/or wild-type YTHDF3 or the YTHDF3 MT6mA mutant immunopurified from ALKBH1 KO HeLa cells was incubated with 6mA-dsDNA oligos, and the binding of ALKBH1 (A1) and YTHDF3 (F3) to 6mA-dsDNA was evaluated. (C, D) The 6mA levels shown in Figure 7D were also determined via dot blotting (C) and 6mA-ELISA (D). (E, F) ALKBH1-Flag immunopurified from YTHDF3 KO cells and/or wild-type YTHDF3-Flag or the YTHDF3-Flag MT6mA mutant immunopurified from ALKBH1 KO HeLa cells was incubated with 6mA-dsDNA oligos, and the 6mA levels were determined by dot botting and LC–MS/MS (E) and 6mA-ELISA (F). (G) ALKBH1-Flag immunopurified from YTHDF3 KO HeLa cells and/or wild-type YTHDF3-Flag or the YTHDF3-Flag MTbind mutant immunopurified from ALKBH1 KO HeLa cells was incubated with 6mA-dsDNA oligos, and the binding of ALKBH1 and YTHDF3 to 6mA-dsDNA was evaluated. (H, I) The 6mA levels shown in Figure 7F were also determined via dot blotting (H) and 6mA-ELISA (I). (J, K) ALKBH1 immunopurified from YTHDF3 KO cells and/or wild-type YTHDF3 or the YTHDF3 MTbind mutant immunopurified from ALKBH1 KO HeLa cells was incubated with 6mA-dsDNA oligos, and the 6mA levels were determined by dot botting and LC–MS/MS (J) and 6mA-ELISA (K). Data information: In (D, F), data are presented as mean ± SD. n=3 independent biological replicates, *p<0.05, **p<0.01, ***p<0.001, ns, non-significant (Student's t-test).

Appendix Tables

Appendix Table S1. The 6mA and A-DNA oligonucleotides used in this study.

Oligo names	Oilgo sequences (5'-3')
Bulge-6mA	Sense:<br>5'-AACTTCGTGCAGGCATGGG(6mA)TCTTGTCTACT-3'<br>Antisense:<br>5'-AGTAGACACATGCCTGCACGAAGTT-3'
Bubble-6mA	Sense:<br>5'-AACTTCGTGCAGGCATGGG(6mA)TCTTGTCTACT-3'<br>Antisense:<br>5'-AGTAGACAGTCGATCATGCCTGCACGAAGTT-3'
dsDNA-6mA/6mA-dsDNA	Sense:<br>5'-AACTTCGTGCAGGCATGGG(6mA)TCTTGTCTACT-3'<br>Antisense:<br>5'-AGTAGACAAGATCCCATGCCTGCACGAAGTT-3'
ssDNA-6mA/6mA-ssDNA	sense:<br>5'-AACTTCGTGCAGGCATGGG(6mA)TCTTGTCTACT-3'
M2-dsDNA-A	Sense:<br>5'-TGCAGGAAACACGAGATTTCATGAAGCAGTATTCAG<br>TCATCAAGTGATGCAGAGCTTGTATAGAAGATCGACTA<br>GAAATCATCTTCATGAAGAGTGATTTTGGCACAAGTGA<br>CCGAAGAACAAAACACCA-3'<br>Antisense:<br>5'-TGGTGTTTTGTTCTTCGGTCACTTGTGCCAAAATCAC<br>TCTTCATGAAGATGATTTCTAGTCGATCTTCTATACAAG

	CTCTGCATCACTTGATGACTGAATACTGCTTCATGAAAT CTCGTGTTTCCTGCA-3’
M2-dsDNA-6mA	Sense: 5’-TGCAGGAAACACGAGATTTCATGAAGCAGTATTCAG TCATCAAGTGATGCAGAGCTTGTATAGAAG(6mA)TCGA CTAGAAATCATCTTCATGAAGAGTGATTTTGGCACAAG TGACCGAAGAACAAAACACCA-3’ Antisense: 5’-TGGTGTTTTGTTCTTCGGTCACTTGTGCCAAAATCAC TCTTCATGAAGATGATTTCTAGTCGATCTTCTATACAAG CTCTGCATCACTTGATGACTGAATACTGCTTCATGAAAT CTCGTGTTTCCTGCA-3’
M3-dsDNA-A	Sense: 5’-TCTGGCCCTCTGAGATGACTATTTTGCGTGGGACAGG GTTGATCTGAATATAGGTATTTGCAGAAAAAGCGATC GTTTGGTAAATTCAAAGTTTATTGATTTAGAGTGGGTTT CTTCACCTTGGCACTA-3’ Antisense: 5’-TAGTGCCAAGGTGAAGAAACCCACTCTAAATCAATA AACTTTGAATTTACCAAACGATCGCTTTTTCTGCAAAT ACCTATATTCAGATCAACCCTGTCCCACGCAAAATAGT CATCTCAGAGGGCCAGA-3’

M3-dsDNA-6mA	Sense: 5’-TCTGGCCCTCTGAGATGACTATTTTGCGTGGGACAGGGTTG(6mA)TCTGAATATAGGTATTTGCAGAAAAAAGCG(6mA)TCGTTTGGTAAATTCAAAGTTTATTGATTTAGAGTGGGTTTCTTCACCTTGGCACTA-3’ Antisense: 5’-TAGTGCCAAGGTGAAGAAACCCACTCTAAATCAATAAACTTTGAATTTACCAAACGATCGCTTTTTTCTGCAAATACCTATATTCAGATCAACCCTGTCCCACGCAAAATAGTCATCTCAGAGGGCCAGA-3’
6mA-bulge-DNA (for enzyme kinetics assay)	Sense: 5’-ATTTCATGAAGCAGTATTCAGTCATCAAGTGATGCAGAGCTTGTATAGAAG(6mA)TCGACTAGAAATCATCTTCATGAAGAGTGATTTTGGCACAAGTGACCG-3’ Antisense: 5’-CGGTCACTTGTGCCAAAATCACTCTTCATGAAGATGATTTCTAGTTCTATACAAGCTCTGCATCACTTGATGACTGAATACTGCTTCATGAAAT-3’
6mA-bubble-DNA (for enzyme kinetics assay)	Sense: 5’-ATTTCATGAAGCAGTATTCAGTCATCAAGTGATGCAGAGCTTGTATAGAAG(6mA)TCGACTAGAAATCATCTTCATGAAGAGTGATTTTGGCACAAGTGACCG-3’

	Antisense:<br>5'-CGGTCACTTGTGCCAAAATCACTCTTCATGAAGATG<br>ATTTCTAGTTTCGACTCTATACAAGCTCTGCATCACTTG<br>ATGACTGAATACTGCTTCATGAAAT-3'
6mA-dsDNA (for enzyme kinetics assay)	Sense:<br>5'-ATTTCATGAAGCAGTATTCAGTCATCAAGTGATGCA<br>GAGCTTGTATAGAAG(6mA)TCGACTAGAAATCATCTTC<br>ATGAAGAGTGATTTTGGCACAAGTGACCG-3'<br>Antisense:<br>5'-CGGTCACTTGTGCCAAAATCACTCTTCATGAAGATG<br>ATTTCTAGTCGATCTTCTATACAAGCTCTGCATCACTTG<br>ATGACTGAATACTGCTTCATGAAAT-3'
6mA-ssDNA (for enzyme kinetics assay)	Sense:<br>5'-ATTTCATGAAGCAGTATTCAGTCATCAAGTGATGCA<br>GAGCTTGTATAGAAG(6mA)TCGACTAGAAATCATCTTC<br>ATGAAGAGTGATTTTGGCACAAGTGACCG-3'

Appendix Table S2. The siRNA sequences used in this study.

Gene name	siRNA No.	siRNA sequences
ALKBH1	si#1	Sense: 5’-GCAAGCCUAUGGACUCAAATT-3’<br>Antisense: 5’-UUUGAGUCCAUAGGGCUUGCTT-3’
YTHDF3	si#1	sense: 5’- GGGACAAUCAACACAAAGUTT -3’<br>antisense: 5’- ACUUUGUGUUGAUUGUCCCTT -3’
	si#2	sense: 5’- GGACGUGUGUUUAUAAUUATT -3’<br>antisense: 5’- UAAUUAUAAACACACGUCCTT -3’
MTHFD1	si#1	sense: 5’- GCAACAGAGAUGAUUCCAATT -3’<br>antisense: 5’- UUGGAAUCAUCUCUGUUGCTT -3’
	si#2	sense: 5’- GCAACUGGUCAGCCUGAAATT -3’<br>antisense: 5’- UUUCAGGCUGACCAGUUGCTT -3’
MMS19	si#1	sense: 5’- GCAAGUGAACAGAGGGAAUTT -3’<br>antisense: 5’- AUUCCCUCUGUUCACUUGCTT -3’
	si#2	sense: 5’- GCGGUUCUUCACAGAUAAUTT -3’<br>antisense: 5’- AUUAUCUGUGAAGAACCGCTT -3’
NC		sense: 5’- UUCUCCGAACGUGUCACGUTT -3’<br>antisense: 5’- ACGUGACACGUUCGGAGAATT -3’

Appendix Table S3. The RT-PCR and qPCR primers used in this study.

Primers name		Sequence (5'-3')
YTHDF3 (For RT-PCR)	Forward	GCCTATGCCATATCTGACAACC
	Reverse	GCCTGTCCATCAGTAATAGCTC
MTHFD1 (For RT-PCR)	Forward	GTTCTCTGACATCCAAATCCGAA
	Reverse	TGTGTGACCCTTCTCCGTTG
MMS19 (For RT-PCR)	Forward	GACTATAGCCTGGGACCCTT
	Reverse	CAACTTGGCACTCAGAACCTC
GAPDH (For RT-PCR)	Forward	TCTTCCAGGAGCGAGATCCCT
	Reverse	TGGTCATGAGTCCTTCCACGAT
M2 (For qPCR)	Forward	TCTATCAGGCTGCAGGAAACA
	Reverse	TTGGCTGCTATGGTGTTTTGT
M3 (For qPCR)	Forward	TCTGGCCCTCTGAGATGACT
	Reverse	CCATGACAGAGAGTTATCCAGC
M4 (For qPCR)	Forward	TCCAGAAAATACGGAGCAGTT
	Reverse	CACAAAATGCCCAAGAAACA
M5 (For qPCR)	Forward	GGAAAAATGATATGGTGTGTTGG
	Reverse	CAACTGCTGCTGGACACATT

GAPDH	Forward	GGGAAACTGTGGCGTGAT
(For qPCR)	Reverse	GAGTGGGTGTCGCTGTTGA
M2-oligo	Forward	TGCAGGAAACACGAGATTTC
(For qPCR)	Reverse	TGGTGTTTTGTTCTTCGGTC
M3-oligo	Forward	TCTGGCCCTCTGAGATGACT
(For qPCR)	Reverse	TAGTGCCAAGGTGAAGAAAC
TMEM234	Forward	GCCTAGAGAAGGGACACTGG
(For qPCR)	Reverse	ACTTTGTGAAGACAGACGGT
DCST2	Forward	AACTGGCGCTCTGTGGACTT
(For qPCR)	Reverse	CTCTAATCGTCCTCCTGAGC
CABIN1	Forward	TAGCCAGGCATGGTGGCAGG
(For qPCR)	Reverse	GAGACAGAGTGTCACTCCGTAA
COASY	Forward	AACTTCCTAAGGGCTCCTAA
(For qPCR)	Reverse	TACCTTCTCTAGAAGAGGCAG
KDELR3	Forward	TCTCAAACTCCTGACCTCG
(For qPCR)	Reverse	GAGCTAGATTCTGTCTAAAC
ATG7	Forward	CATTTCCTCCCTTGCTCCCT
(For qPCR)	Reverse	TCTATTTTAGTAAAGGCAAT

AGRN	Forward	TCTACTGTGGACATTTGCCC
(For qPCR)	Reverse	CCACCAGGTCTTTGCCCTTC
SDF4	Forward	ATCCTGGGGTTGCAGGCATG
(For qPCR)	Reverse	TTTAGAACGGACAGGACCAA
PKP1	Forward	TGGGCAGGACTGCTAGGTAC
(For qPCR)	Reverse	GCCAAGCCCAGAATGACATA
CCNL2	Forward	GGATCTGTGTTGAAAGTCGT
(For qPCR)	Reverse	ATAACGCTCCGGATGTCCTG

Appendix Table S4. The biotin-labeled DNA oligonucleotides containing A or 6mA used in this study.

Oligo names	Oilgo sequences (5'-3')
6mA-ds-oligo	Sense: 5'-CTATGTTTC(6mA)TGTACCGCCA-Biotin-3'<br>Antisense: 5'- TGGCGGTACATGAAACATAG-3'
A-ds-oligo	Sense: 5'-CTATGTTTCATGTACCGCCA-Biotin-3'<br>Antisense: 5'- TGGCGGTACATGAAACATAG-3'
Bulge-6mA	Sense:5'-AACTTCGTGCAGGCATGGG(6mA)TCTTGTCTACT-Biotin-3'<br>Antisense: 5'- AGTAGACACATGCCTGCACGAAGTT-3'
Bubble-6mA	Sense:5'-AACTTCGTGCAGGCATGGG(6mA)TCTTGTCTACT-Biotin-3'<br>Antisense:5'-AGTAGACAGTCGATCATGCCTGCACGAAGTT-3'
ds-6mA	Sense:5'-AACTTCGTGCAGGCATGGG(6mA)TCTTGTCTACT-Biotin-3'<br>Antisense:5'-AGTAGACAAGATCCCATGCCTGCACGAAGTT-3'
ss-6mA	sense:5'-AACTTCGTGCAGGCATGGG(6mA)TCTTGTCTACT--Biotin-3'
Bulge-A	Sense:5'-AACTTCGTGCAGGCATGGGATCTTGTCTACT-Biotin-3'<br>Antisense: 5'- AGTAGACACATGCCTGCACGAAGTT-3'
Bubble-A	Sense:5'-AACTTCGTGCAGGCATGGGATCTTGTCTACT-Biotin-3'<br>Antisense:5'-AGTAGACAGTCGATCATGCCTGCACGAAGTT-3'

ds-A	Sense:5’-AACTTCGTGCAGGCATGGGATCTTGTCTACT-Biotin-3’<br>Antisense:5’-AGTAGACAAGATCCCATGCCTGCACGAAGTT-3’
ss-A	Sense:5’-AACTTCGTGCAGGCATGGGATCTTGTCTACT-Biotin-3’
ss-m^6A-oligo	Sense:5’-Biotin-CGUCUCGG(m^6A)CUCGGACUGCU-3’
tRNA-m^1A-oligo	Sense:5’-CCCGGUUCG(m^1A)UUCCCGG-Biotin-3’
