# Supplementary figures and images for: YTHDF3 recognizes DNA N6-methyladenine and recruits ALKBH1 for 6mA removal from genomic DNA (part 1 of 2)
Source: EMBO J. 2025 Jul 25;44(17):4899–917. doi: 10.1038/s44318-025-00512-2 (PMC12402098; doi:10.1038/s44318-025-00512-2)

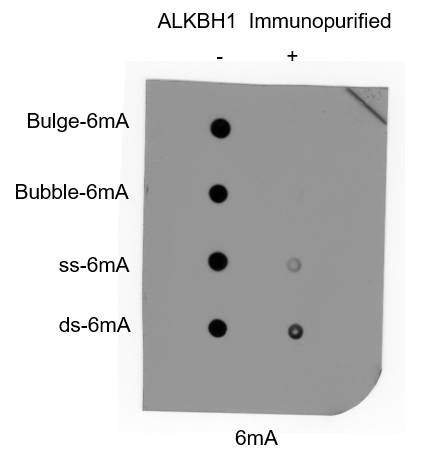

Supplement: Supplementary file 4 — Source data Fig. 1 [file 44318_2025_512_MOESM4_ESM.zip › Figure_1/1A/ALKBH1 Immunopurified-6mA.tif]

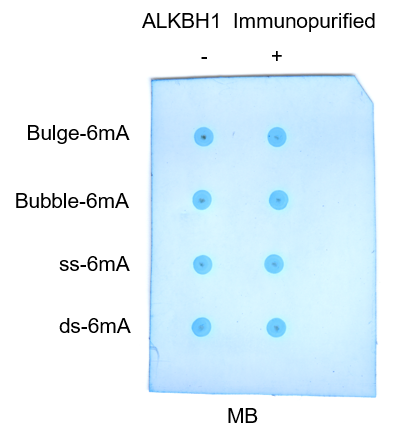

Supplement: Supplementary file 4 — Source data Fig. 1 [file 44318_2025_512_MOESM4_ESM.zip › Figure_1/1A/ALKBH1 Immunopurified-MB.tif]

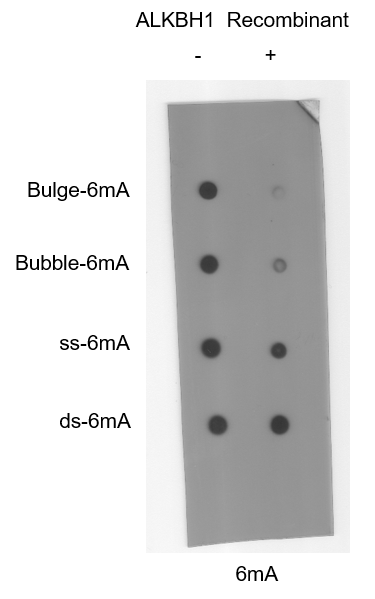

Supplement: Supplementary file 4 — Source data Fig. 1 [file 44318_2025_512_MOESM4_ESM.zip › Figure_1/1A/ALKBH1 Recombinant-6mA.tif]

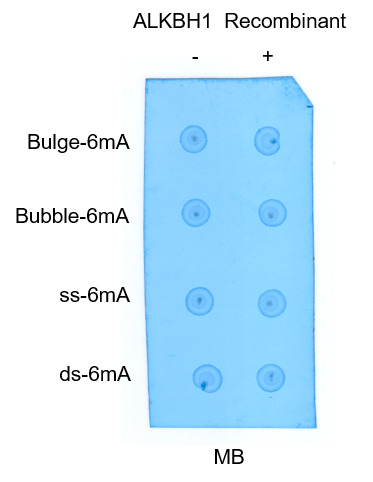

Supplement: Supplementary file 4 — Source data Fig. 1 [file 44318_2025_512_MOESM4_ESM.zip › Figure_1/1A/ALKBH1 Recombinant-MB.tif]

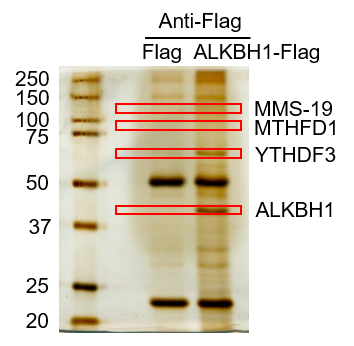

Supplement: Supplementary file 4 — Source data Fig. 1 [file 44318_2025_512_MOESM4_ESM.zip › Figure_1/1D/Gel stained with silver.tif]

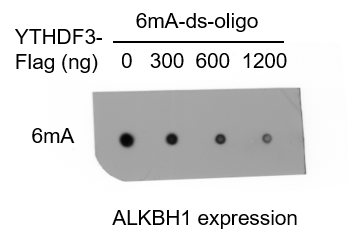

Supplement: Supplementary file 4 — Source data Fig. 1 [file 44318_2025_512_MOESM4_ESM.zip › Figure_1/1G/(ALKBH1-expression) YTHDF3-6mA.tif]

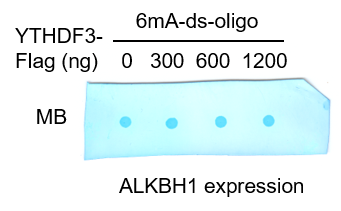

Supplement: Supplementary file 4 — Source data Fig. 1 [file 44318_2025_512_MOESM4_ESM.zip › Figure_1/1G/(ALKBH1-expression) YTHDF3-MB.tif]

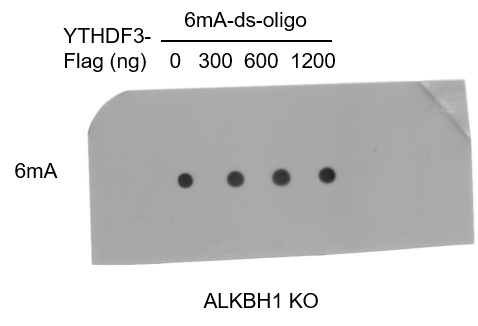

Supplement: Supplementary file 4 — Source data Fig. 1 [file 44318_2025_512_MOESM4_ESM.zip › Figure_1/1G/(ALKBH1-KO) YTHDF3-6mA.tif]

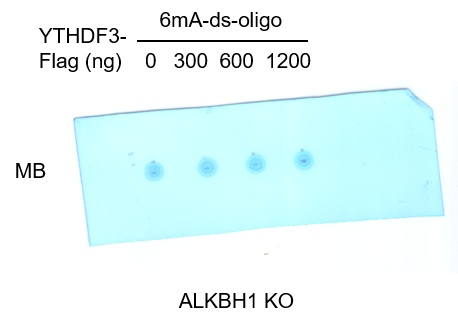

Supplement: Supplementary file 4 — Source data Fig. 1 [file 44318_2025_512_MOESM4_ESM.zip › Figure_1/1G/(ALKBH1-KO) YTHDF3-MB.tif]

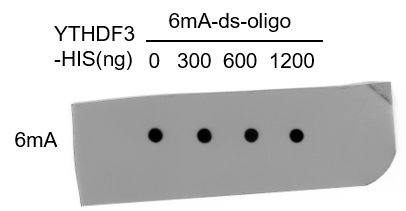

Supplement: Supplementary file 4 — Source data Fig. 1 [file 44318_2025_512_MOESM4_ESM.zip › Figure_1/1H/F3-His 6mA.tif]

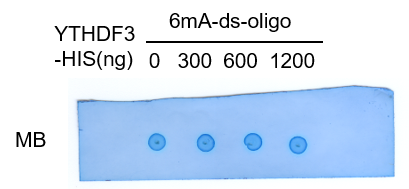

Supplement: Supplementary file 4 — Source data Fig. 1 [file 44318_2025_512_MOESM4_ESM.zip › Figure_1/1H/F3-His MB.tif]

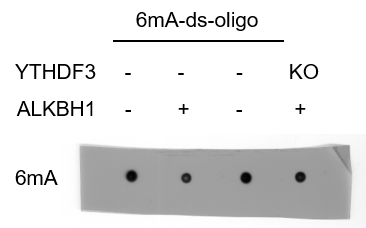

Supplement: Supplementary file 5 — Source data Fig. 2 [file 44318_2025_512_MOESM5_ESM.zip › Figure_2/2C (old panel S2E)/(6mA) A1-Flag VS KO-F3+A1-Flag.tif]

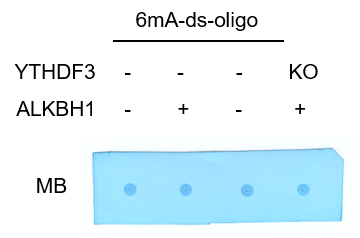

Supplement: Supplementary file 5 — Source data Fig. 2 [file 44318_2025_512_MOESM5_ESM.zip › Figure_2/2C (old panel S2E)/(MB) A1-Flag VS KO-F3+A1-Flag.tif]

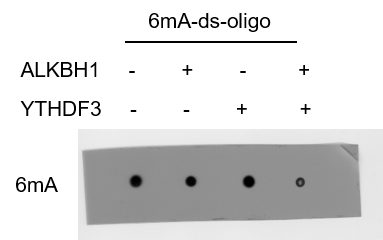

Supplement: Supplementary file 5 — Source data Fig. 2 [file 44318_2025_512_MOESM5_ESM.zip › Figure_2/2D (old panel S2F)/(6mA) A1-Flag(KO-F3)+F3-Flag(KO-A1).tif]

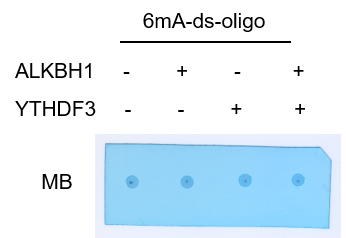

Supplement: Supplementary file 5 — Source data Fig. 2 [file 44318_2025_512_MOESM5_ESM.zip › Figure_2/2D (old panel S2F)/(MB) A1-Flag(KO-F3)+F3-Flag(KO-A1).tif]

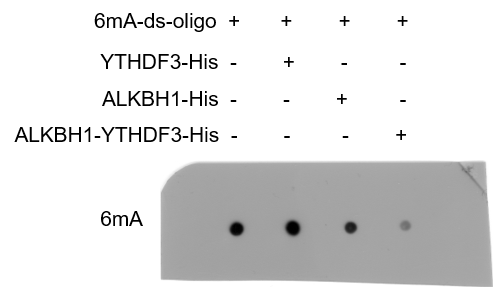

Supplement: Supplementary file 5 — Source data Fig. 2 [file 44318_2025_512_MOESM5_ESM.zip › Figure_2/2G/6mA.tif]

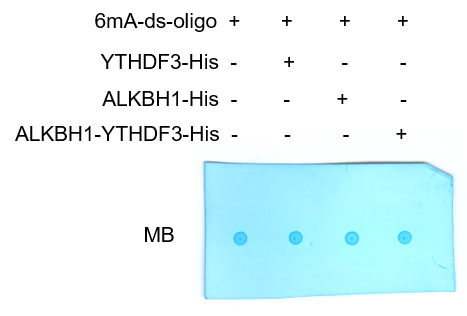

Supplement: Supplementary file 5 — Source data Fig. 2 [file 44318_2025_512_MOESM5_ESM.zip › Figure_2/2G/MB.tif]

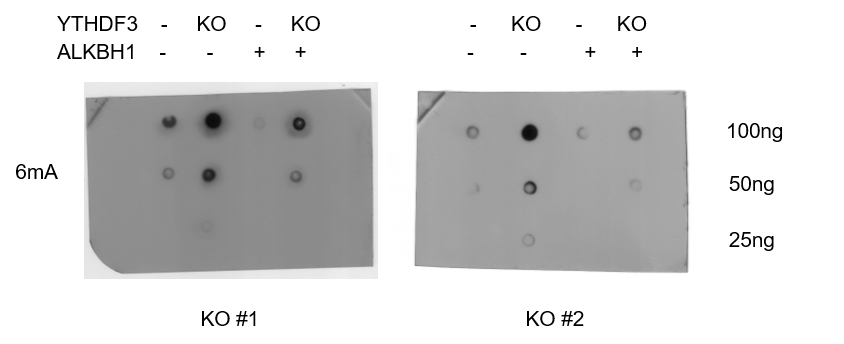

Supplement: Supplementary file 5 — Source data Fig. 2 [file 44318_2025_512_MOESM5_ESM.zip › Figure_2/S2A (old panel 2A)/6mA.tif]

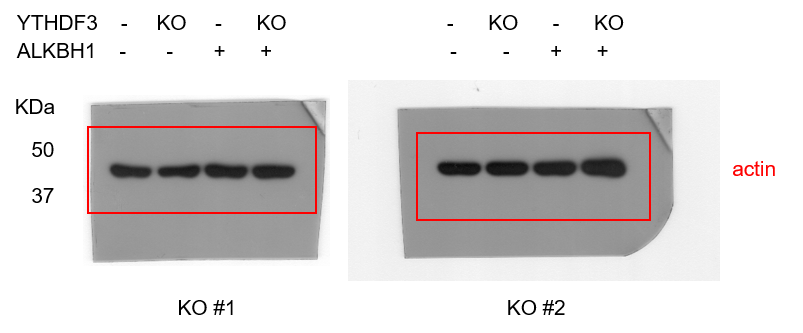

Supplement: Supplementary file 5 — Source data Fig. 2 [file 44318_2025_512_MOESM5_ESM.zip › Figure_2/S2A (old panel 2A)/actin.tif]

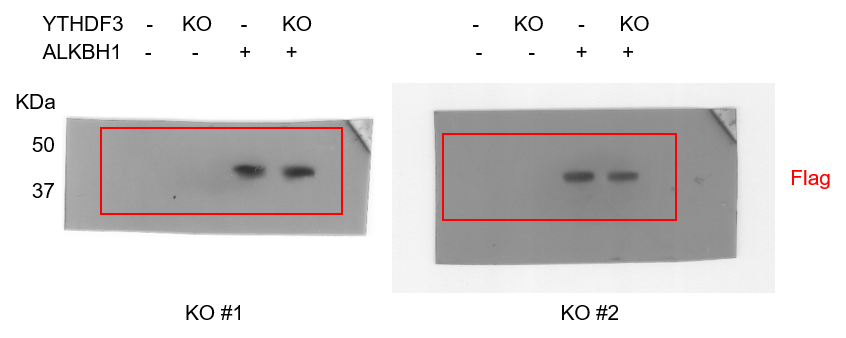

Supplement: Supplementary file 5 — Source data Fig. 2 [file 44318_2025_512_MOESM5_ESM.zip › Figure_2/S2A (old panel 2A)/Flag.tif]

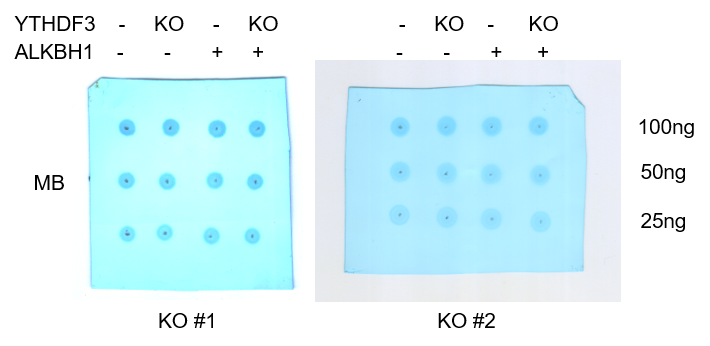

Supplement: Supplementary file 5 — Source data Fig. 2 [file 44318_2025_512_MOESM5_ESM.zip › Figure_2/S2A (old panel 2A)/MB.tif]

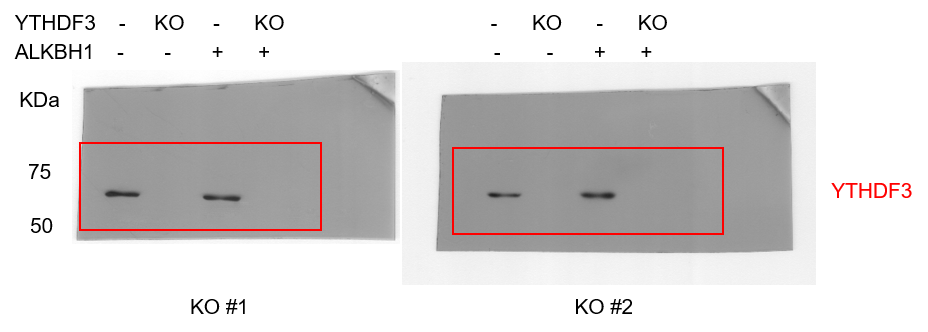

Supplement: Supplementary file 5 — Source data Fig. 2 [file 44318_2025_512_MOESM5_ESM.zip › Figure_2/S2A (old panel 2A)/YTHDF3.tif]

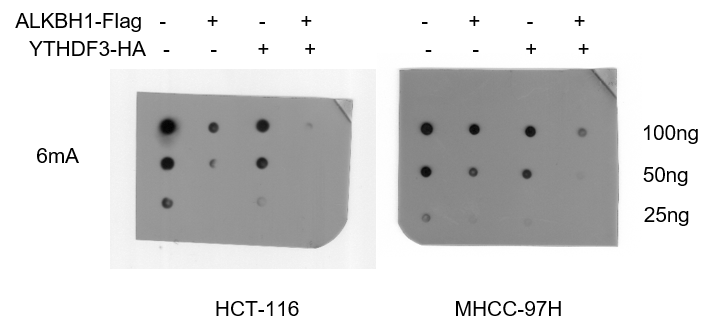

Supplement: Supplementary file 5 — Source data Fig. 2 [file 44318_2025_512_MOESM5_ESM.zip › Figure_2/S2D (old panel 2C)/6mA.tif]

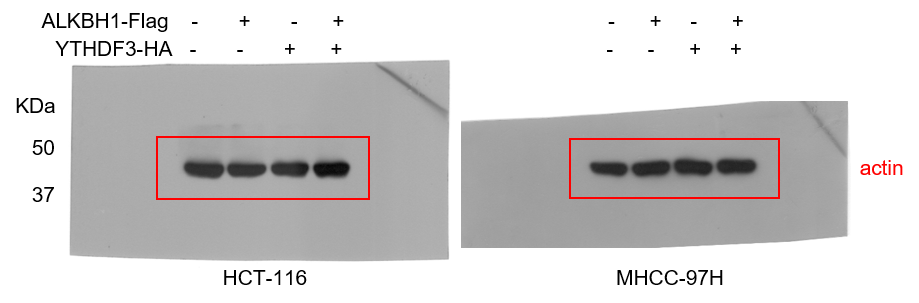

Supplement: Supplementary file 5 — Source data Fig. 2 [file 44318_2025_512_MOESM5_ESM.zip › Figure_2/S2D (old panel 2C)/actin.tif]

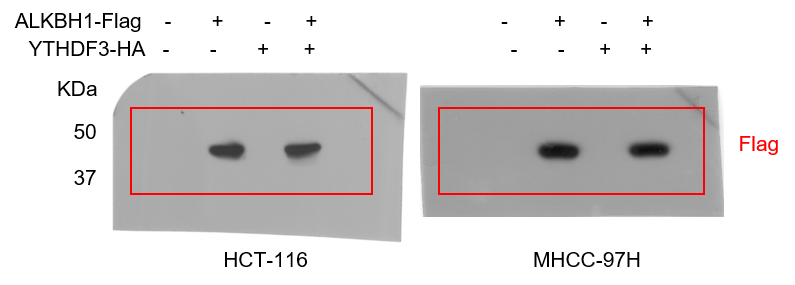

Supplement: Supplementary file 5 — Source data Fig. 2 [file 44318_2025_512_MOESM5_ESM.zip › Figure_2/S2D (old panel 2C)/Flag.tif]

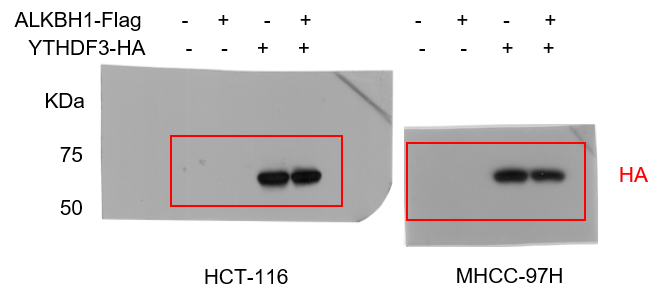

Supplement: Supplementary file 5 — Source data Fig. 2 [file 44318_2025_512_MOESM5_ESM.zip › Figure_2/S2D (old panel 2C)/HA.tif]

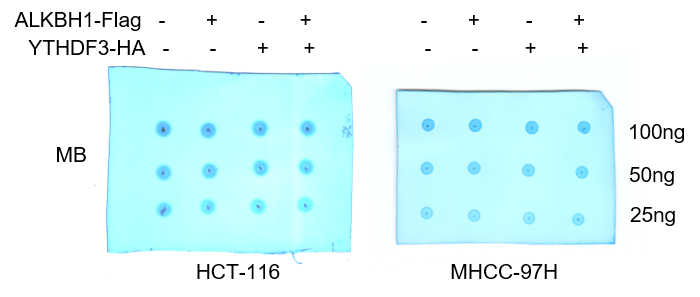

Supplement: Supplementary file 5 — Source data Fig. 2 [file 44318_2025_512_MOESM5_ESM.zip › Figure_2/S2D (old panel 2C)/MB.tif]

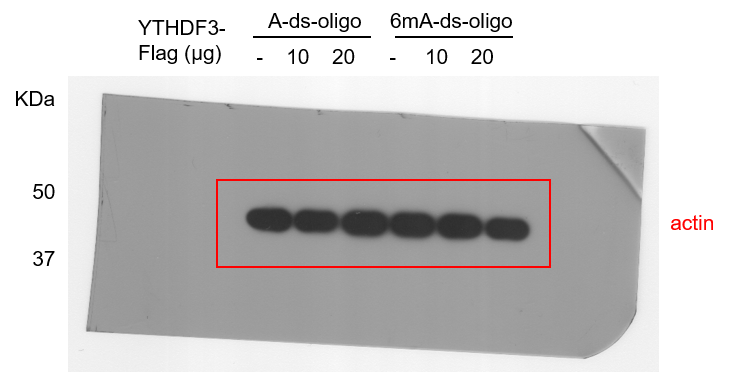

Supplement: Supplementary file 6 — Source data Fig. 3 [file 44318_2025_512_MOESM6_ESM.zip › Figure_3/3A/actin-Total proteins .tif]

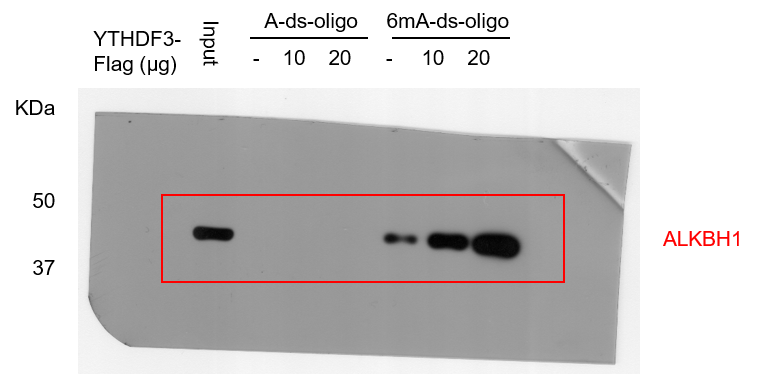

Supplement: Supplementary file 6 — Source data Fig. 3 [file 44318_2025_512_MOESM6_ESM.zip › Figure_3/3A/ALKBH1-DNA pulldown.tif]

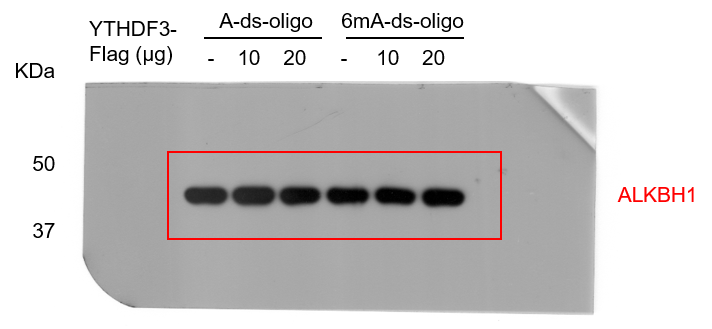

Supplement: Supplementary file 6 — Source data Fig. 3 [file 44318_2025_512_MOESM6_ESM.zip › Figure_3/3A/ALKBH1-Total proteins .tif]

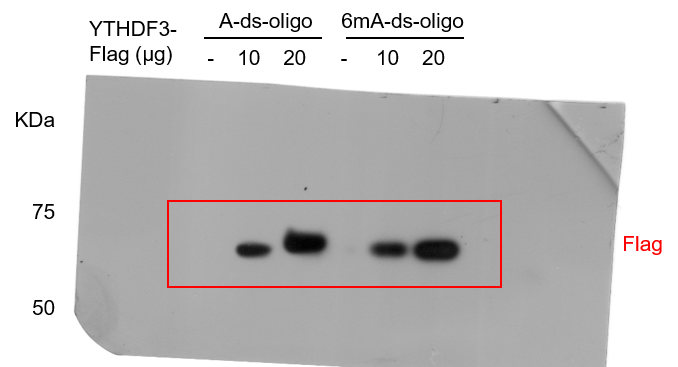

Supplement: Supplementary file 6 — Source data Fig. 3 [file 44318_2025_512_MOESM6_ESM.zip › Figure_3/3A/Flag-Total proteins .tif]

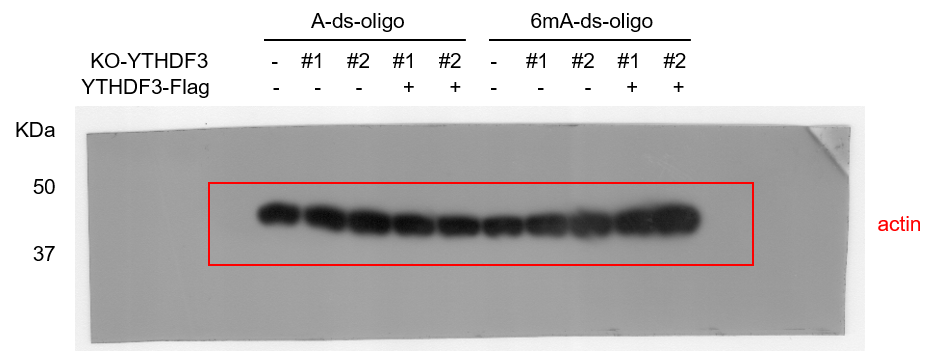

Supplement: Supplementary file 6 — Source data Fig. 3 [file 44318_2025_512_MOESM6_ESM.zip › Figure_3/3B/actin -Total proteins.tif]

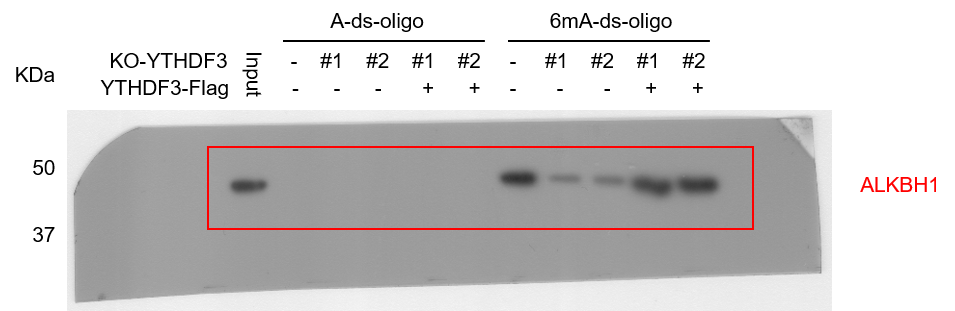

Supplement: Supplementary file 6 — Source data Fig. 3 [file 44318_2025_512_MOESM6_ESM.zip › Figure_3/3B/ALKBH1-Pulldown.tif]

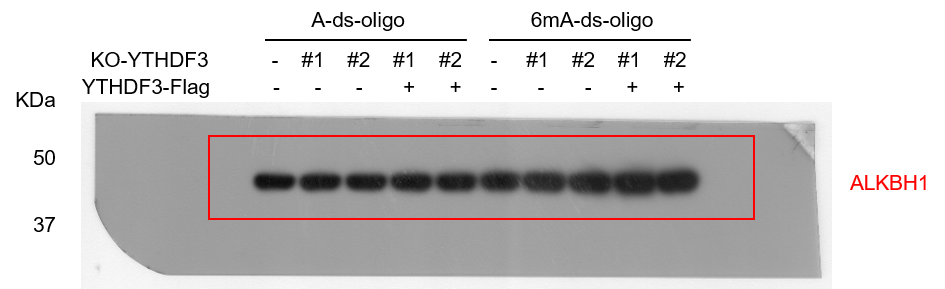

Supplement: Supplementary file 6 — Source data Fig. 3 [file 44318_2025_512_MOESM6_ESM.zip › Figure_3/3B/ALKBH1-Total proteins.tif]

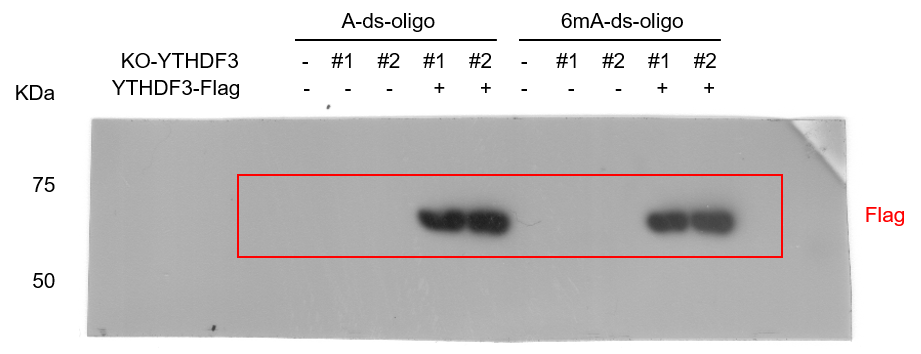

Supplement: Supplementary file 6 — Source data Fig. 3 [file 44318_2025_512_MOESM6_ESM.zip › Figure_3/3B/Flag-Total proteins.tif]

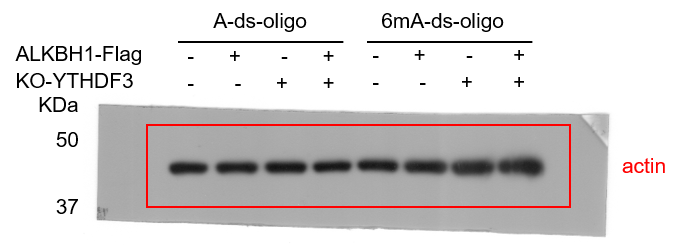

Supplement: Supplementary file 6 — Source data Fig. 3 [file 44318_2025_512_MOESM6_ESM.zip › Figure_3/3C/actin - Total proteins.tif]

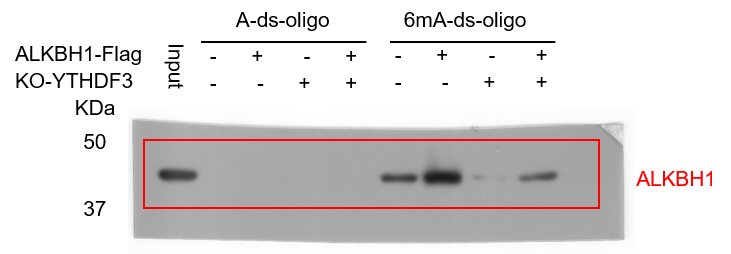

Supplement: Supplementary file 6 — Source data Fig. 3 [file 44318_2025_512_MOESM6_ESM.zip › Figure_3/3C/ALKBH1- pull down.tif]

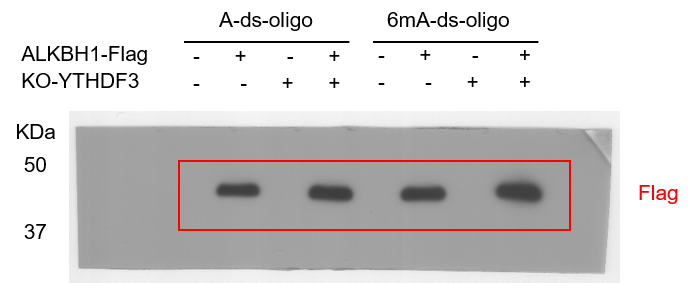

Supplement: Supplementary file 6 — Source data Fig. 3 [file 44318_2025_512_MOESM6_ESM.zip › Figure_3/3C/Flag-- Total proteins.tif]

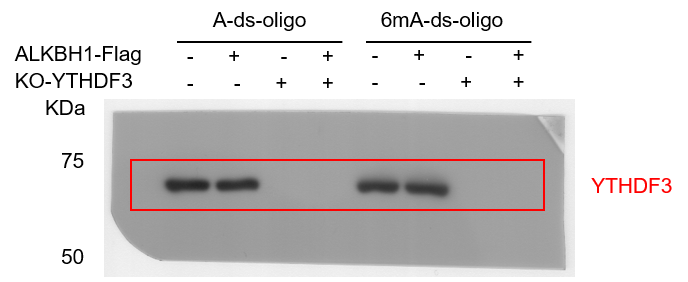

Supplement: Supplementary file 6 — Source data Fig. 3 [file 44318_2025_512_MOESM6_ESM.zip › Figure_3/3C/YTHDF3 - Total proteins.tif]

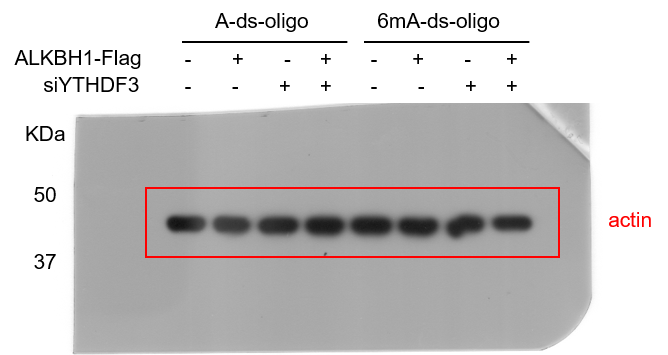

Supplement: Supplementary file 6 — Source data Fig. 3 [file 44318_2025_512_MOESM6_ESM.zip › Figure_3/3D/actin- Total proteins.tif]

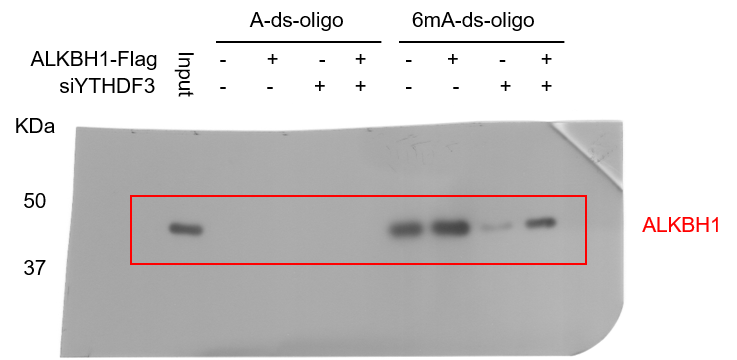

Supplement: Supplementary file 6 — Source data Fig. 3 [file 44318_2025_512_MOESM6_ESM.zip › Figure_3/3D/ALKBH1- pulldown.tif]

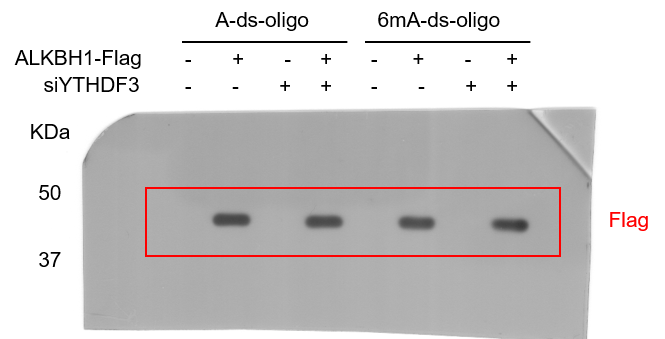

Supplement: Supplementary file 6 — Source data Fig. 3 [file 44318_2025_512_MOESM6_ESM.zip › Figure_3/3D/Flag- Total proteins.tif]

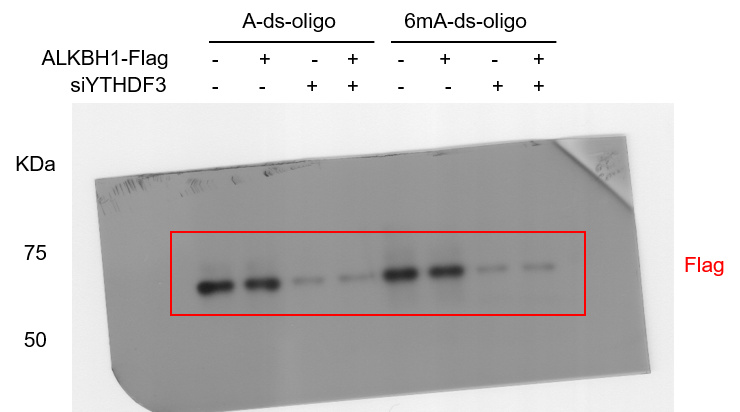

Supplement: Supplementary file 6 — Source data Fig. 3 [file 44318_2025_512_MOESM6_ESM.zip › Figure_3/3D/YTHDF3- Total proteins.tif]

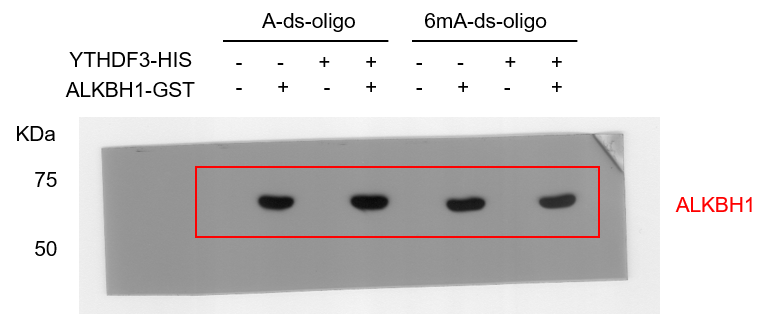

Supplement: Supplementary file 6 — Source data Fig. 3 [file 44318_2025_512_MOESM6_ESM.zip › Figure_3/3E/ALKBH1-Input.tif]

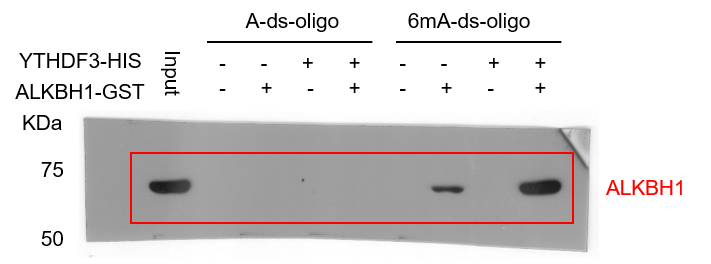

Supplement: Supplementary file 6 — Source data Fig. 3 [file 44318_2025_512_MOESM6_ESM.zip › Figure_3/3E/ALKBH1.tif]

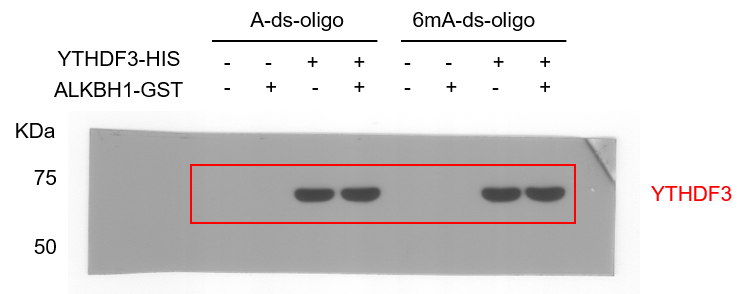

Supplement: Supplementary file 6 — Source data Fig. 3 [file 44318_2025_512_MOESM6_ESM.zip › Figure_3/3E/YTHDF3-Input.tif]

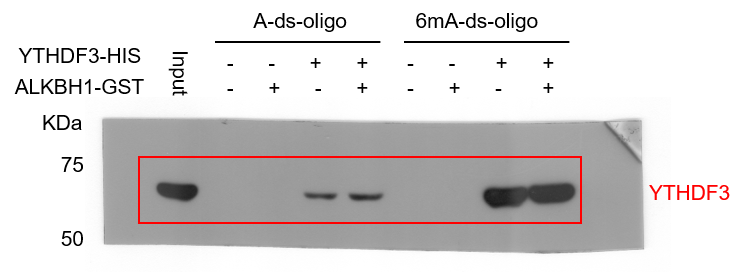

Supplement: Supplementary file 6 — Source data Fig. 3 [file 44318_2025_512_MOESM6_ESM.zip › Figure_3/3E/YTHDF3.tif]

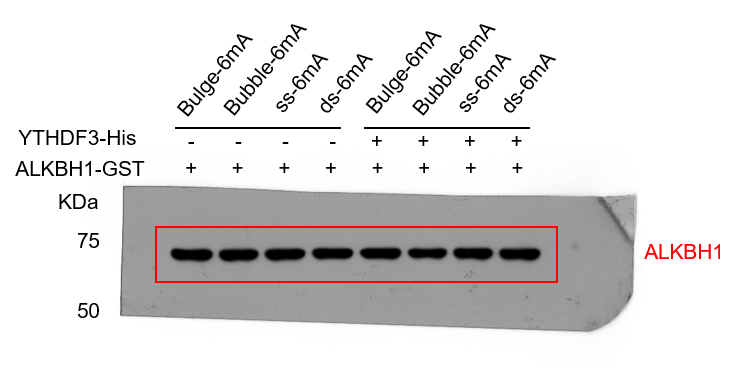

Supplement: Supplementary file 6 — Source data Fig. 3 [file 44318_2025_512_MOESM6_ESM.zip › Figure_3/3F/ALKBH1-Input.tif]

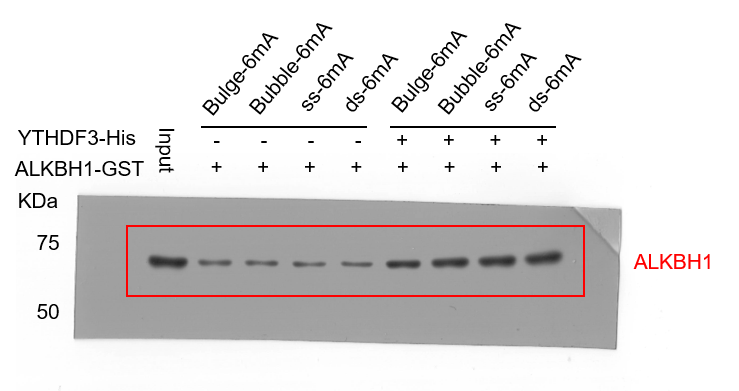

Supplement: Supplementary file 6 — Source data Fig. 3 [file 44318_2025_512_MOESM6_ESM.zip › Figure_3/3F/ALKBH1.tif]

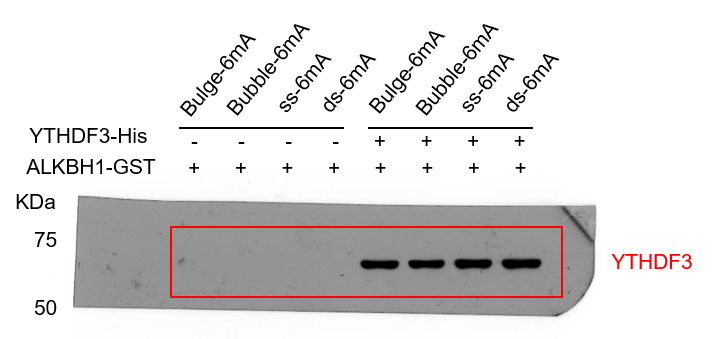

Supplement: Supplementary file 6 — Source data Fig. 3 [file 44318_2025_512_MOESM6_ESM.zip › Figure_3/3F/YTHDF3-Input.tif]

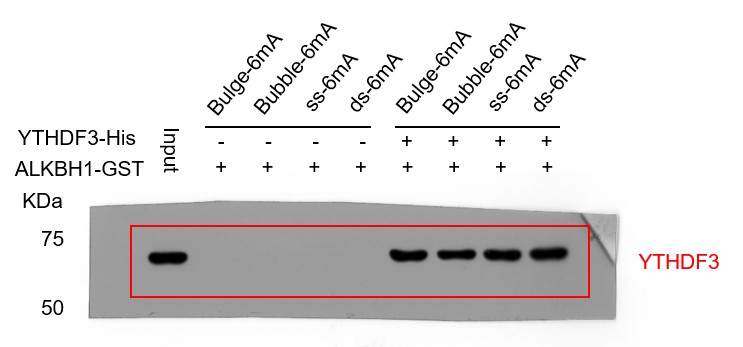

Supplement: Supplementary file 6 — Source data Fig. 3 [file 44318_2025_512_MOESM6_ESM.zip › Figure_3/3F/YTHDF3.tif]

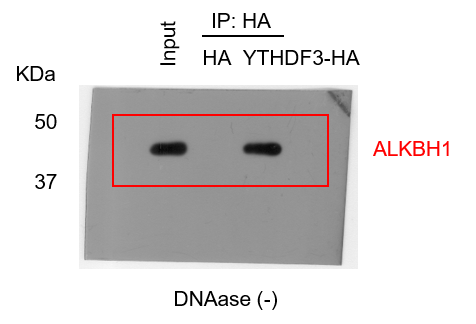

Supplement: Supplementary file 7 — Source data Fig. 4 [file 44318_2025_512_MOESM7_ESM.zip › Figure_4/4A/ALKBH1.tif]

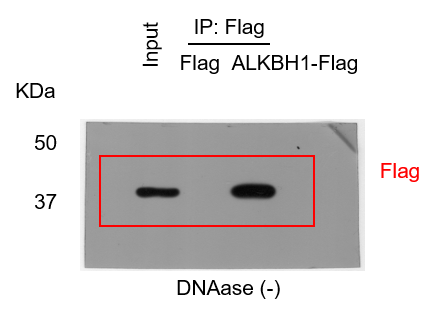

Supplement: Supplementary file 7 — Source data Fig. 4 [file 44318_2025_512_MOESM7_ESM.zip › Figure_4/4A/Flag.tif]

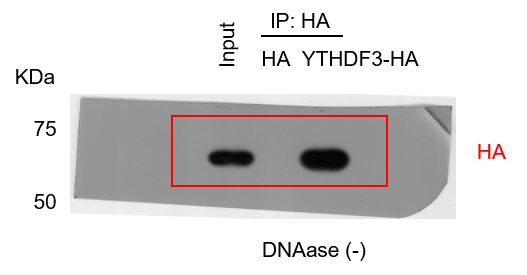

Supplement: Supplementary file 7 — Source data Fig. 4 [file 44318_2025_512_MOESM7_ESM.zip › Figure_4/4A/HA.tif]

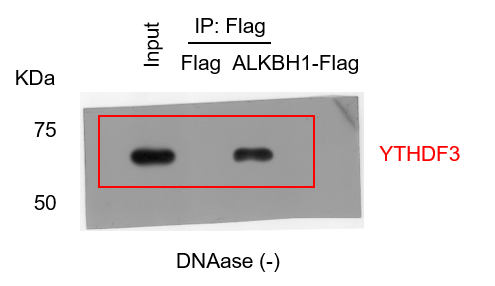

Supplement: Supplementary file 7 — Source data Fig. 4 [file 44318_2025_512_MOESM7_ESM.zip › Figure_4/4A/YTHDF3.tif]

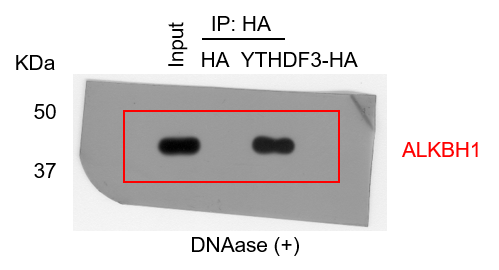

Supplement: Supplementary file 7 — Source data Fig. 4 [file 44318_2025_512_MOESM7_ESM.zip › Figure_4/4B/ALKBH1.tif]

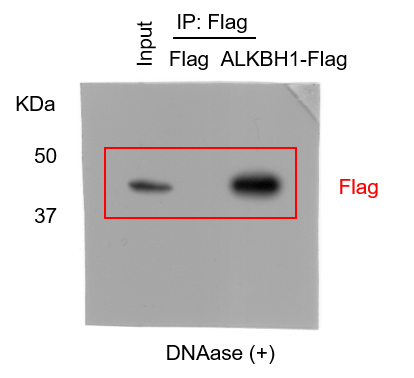

Supplement: Supplementary file 7 — Source data Fig. 4 [file 44318_2025_512_MOESM7_ESM.zip › Figure_4/4B/Flag.tif]

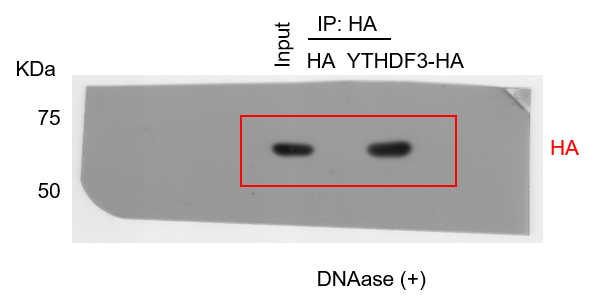

Supplement: Supplementary file 7 — Source data Fig. 4 [file 44318_2025_512_MOESM7_ESM.zip › Figure_4/4B/HA.tif]

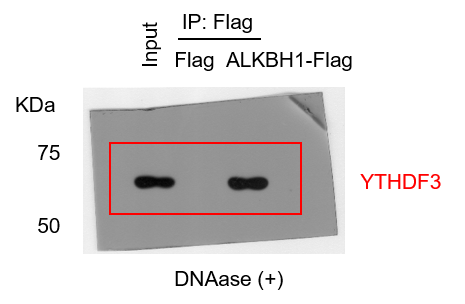

Supplement: Supplementary file 7 — Source data Fig. 4 [file 44318_2025_512_MOESM7_ESM.zip › Figure_4/4B/YTHDF3.tif]

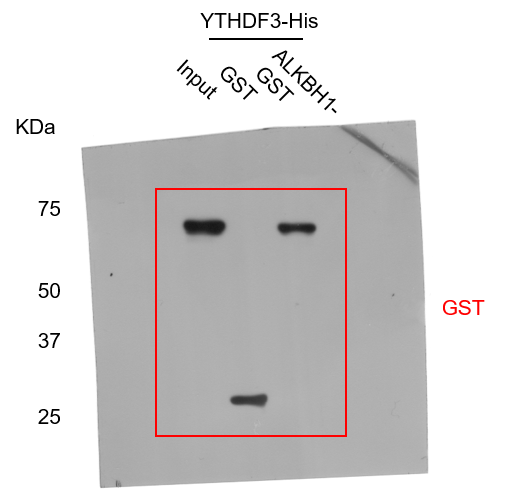

Supplement: Supplementary file 7 — Source data Fig. 4 [file 44318_2025_512_MOESM7_ESM.zip › Figure_4/4C/GST.tif]

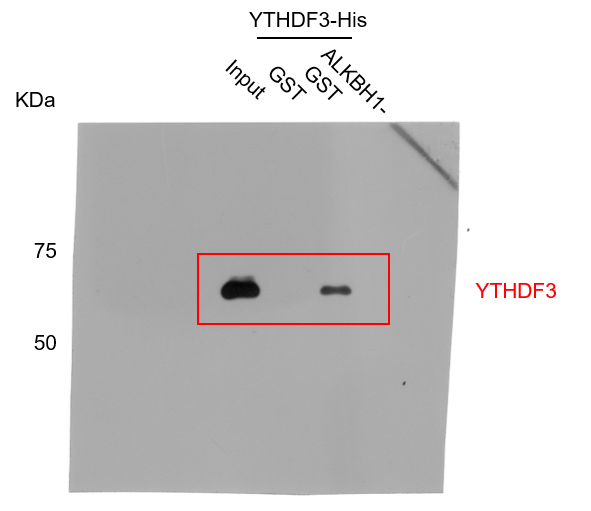

Supplement: Supplementary file 7 — Source data Fig. 4 [file 44318_2025_512_MOESM7_ESM.zip › Figure_4/4C/YTHDF3.tif]

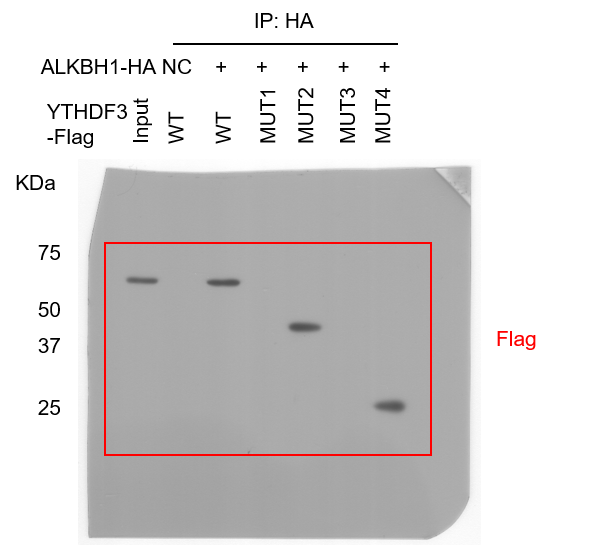

Supplement: Supplementary file 7 — Source data Fig. 4 [file 44318_2025_512_MOESM7_ESM.zip › Figure_4/4E/Flag.tif]

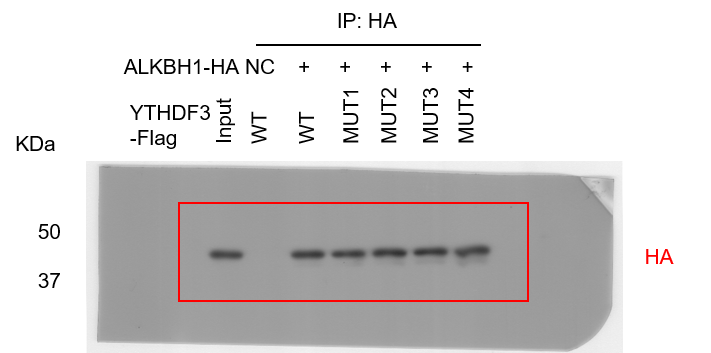

Supplement: Supplementary file 7 — Source data Fig. 4 [file 44318_2025_512_MOESM7_ESM.zip › Figure_4/4E/HA.tif]

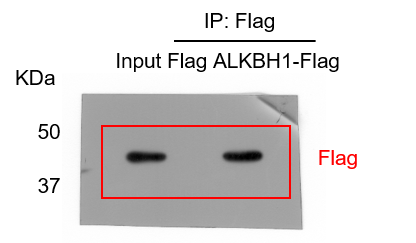

Supplement: Supplementary file 7 — Source data Fig. 4 [file 44318_2025_512_MOESM7_ESM.zip › Figure_4/4F/Flag.tif]

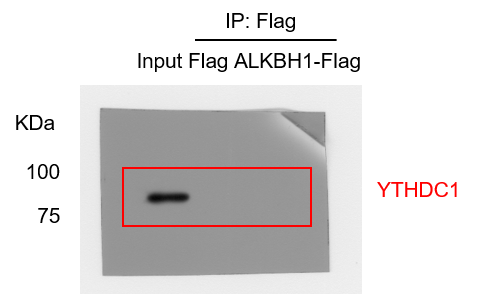

Supplement: Supplementary file 7 — Source data Fig. 4 [file 44318_2025_512_MOESM7_ESM.zip › Figure_4/4F/YTHDC1.tif]

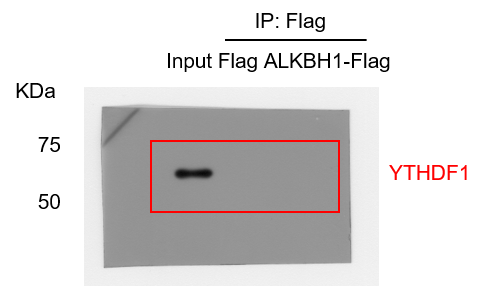

Supplement: Supplementary file 7 — Source data Fig. 4 [file 44318_2025_512_MOESM7_ESM.zip › Figure_4/4F/YTHDF1.tif]

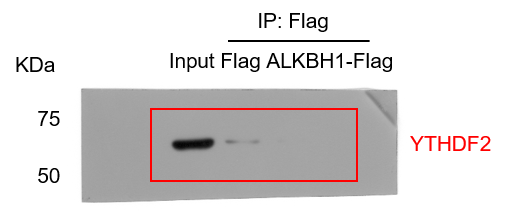

Supplement: Supplementary file 7 — Source data Fig. 4 [file 44318_2025_512_MOESM7_ESM.zip › Figure_4/4F/YTHDF2.tif]

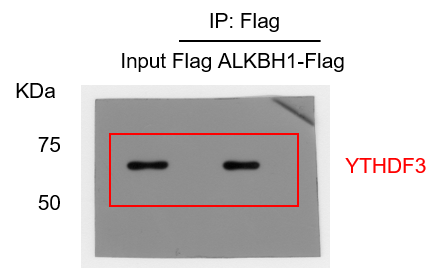

Supplement: Supplementary file 7 — Source data Fig. 4 [file 44318_2025_512_MOESM7_ESM.zip › Figure_4/4F/YTHDF3.tif]

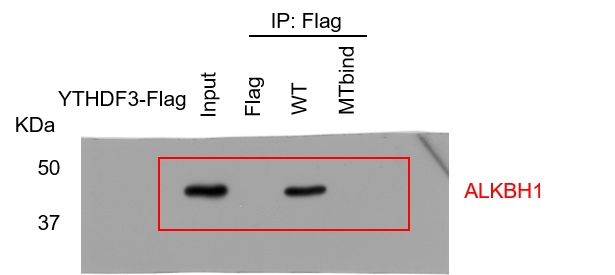

Supplement: Supplementary file 7 — Source data Fig. 4 [file 44318_2025_512_MOESM7_ESM.zip › Figure_4/4H/ALKBH1.tif]

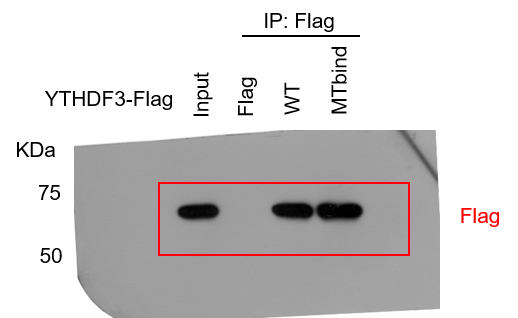

Supplement: Supplementary file 7 — Source data Fig. 4 [file 44318_2025_512_MOESM7_ESM.zip › Figure_4/4H/Flag.tif]

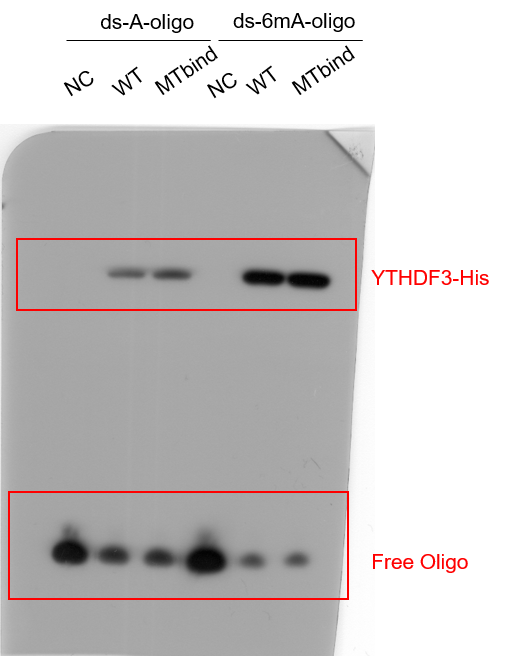

Supplement: Supplementary file 7 — Source data Fig. 4 [file 44318_2025_512_MOESM7_ESM.zip › Figure_4/4I/EMSA.tif]

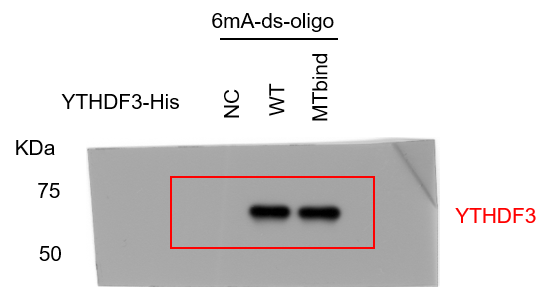

Supplement: Supplementary file 7 — Source data Fig. 4 [file 44318_2025_512_MOESM7_ESM.zip › Figure_4/4J/YTHDF3-INPUT.tif]

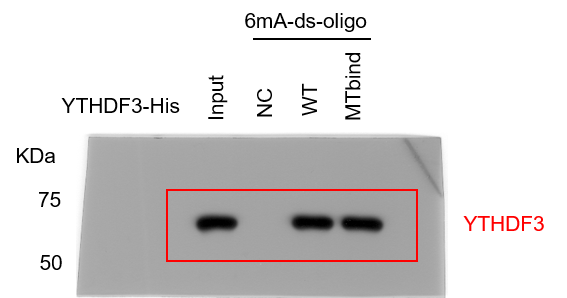

Supplement: Supplementary file 7 — Source data Fig. 4 [file 44318_2025_512_MOESM7_ESM.zip › Figure_4/4J/YTHDF3.tif]

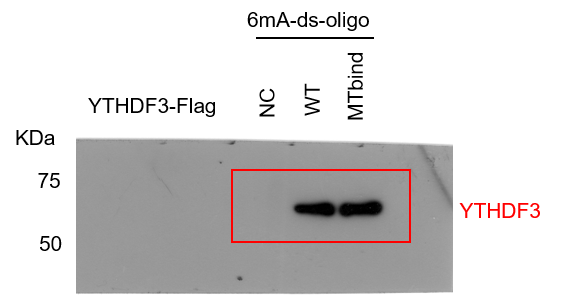

Supplement: Supplementary file 7 — Source data Fig. 4 [file 44318_2025_512_MOESM7_ESM.zip › Figure_4/4K/YTHDF3-INPUT.tif]

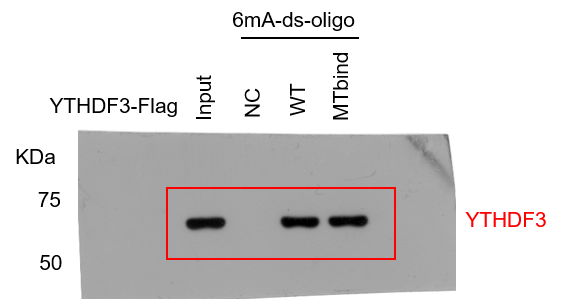

Supplement: Supplementary file 7 — Source data Fig. 4 [file 44318_2025_512_MOESM7_ESM.zip › Figure_4/4K/YTHDF3.tif]

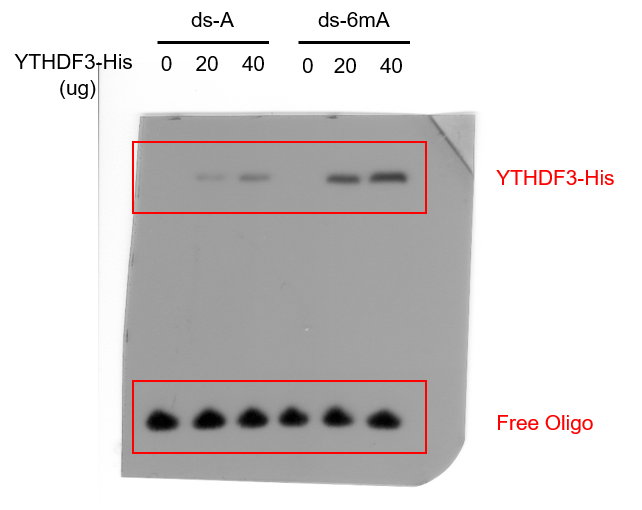

Supplement: Supplementary file 8 — Source data Fig. 5 [file 44318_2025_512_MOESM8_ESM.zip › Figure_5/5A/EMSA.tif]

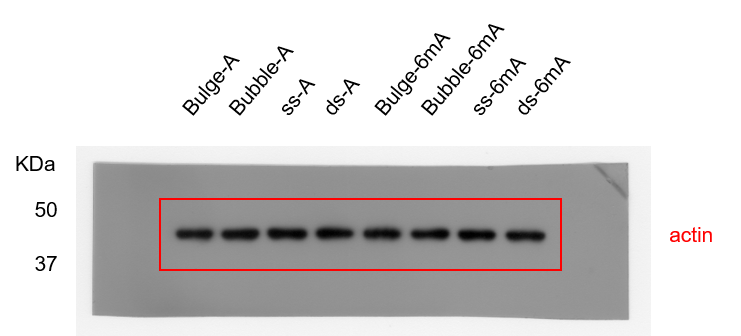

Supplement: Supplementary file 8 — Source data Fig. 5 [file 44318_2025_512_MOESM8_ESM.zip › Figure_5/5B/actin-Total proteins.tif]

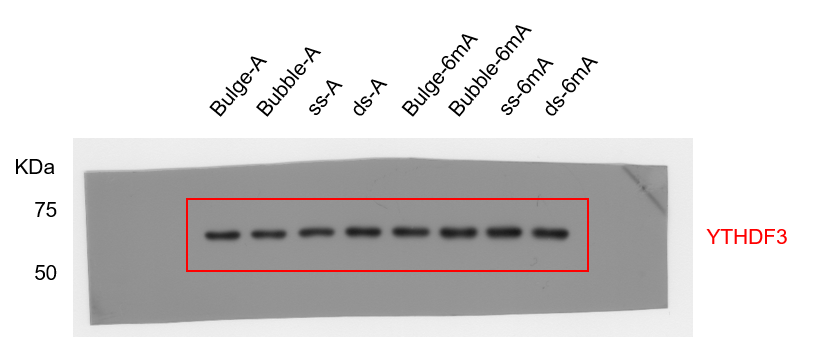

Supplement: Supplementary file 8 — Source data Fig. 5 [file 44318_2025_512_MOESM8_ESM.zip › Figure_5/5B/YTHDF3-Total proteins.tif]

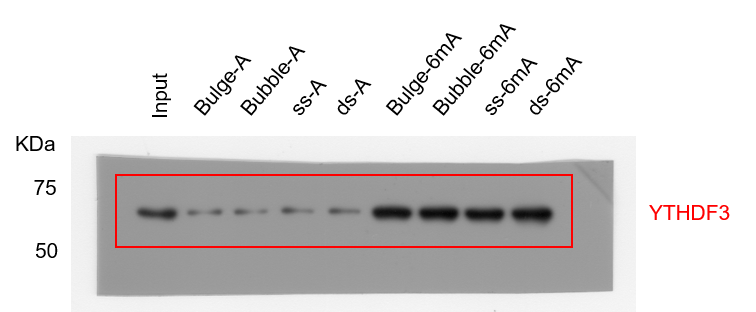

Supplement: Supplementary file 8 — Source data Fig. 5 [file 44318_2025_512_MOESM8_ESM.zip › Figure_5/5B/YTHDF3.tif]

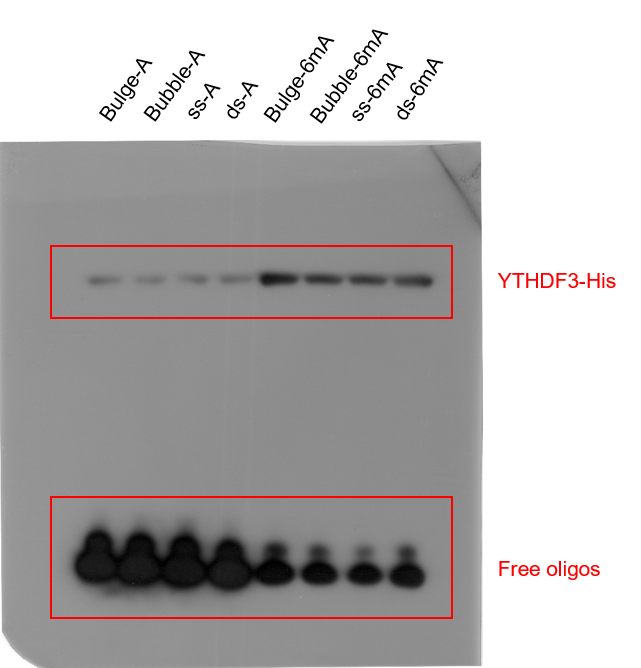

Supplement: Supplementary file 8 — Source data Fig. 5 [file 44318_2025_512_MOESM8_ESM.zip › Figure_5/5C/EMSA.tif]

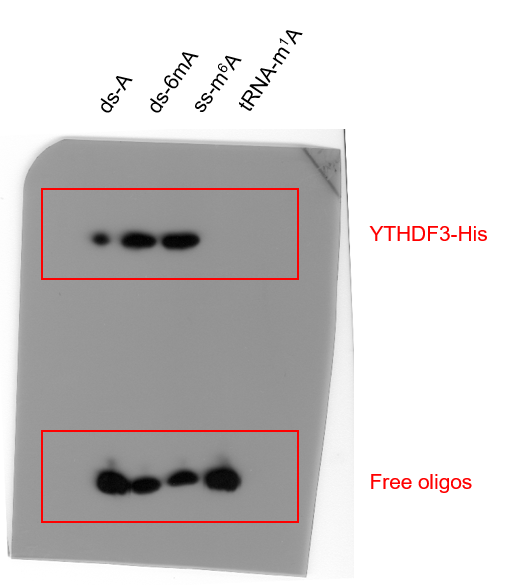

Supplement: Supplementary file 8 — Source data Fig. 5 [file 44318_2025_512_MOESM8_ESM.zip › Figure_5/5D/EMSA.tif]

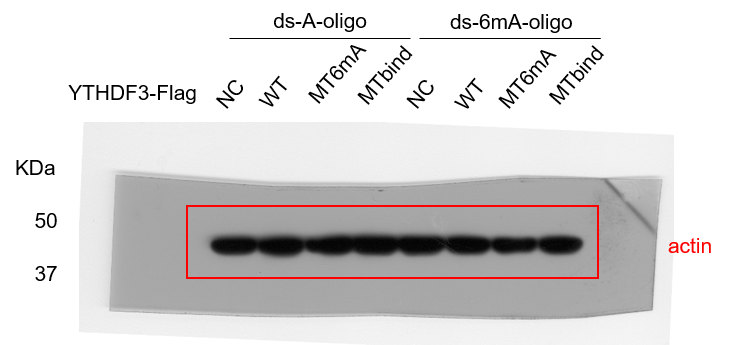

Supplement: Supplementary file 8 — Source data Fig. 5 [file 44318_2025_512_MOESM8_ESM.zip › Figure_5/5F/actin-Total proteins.tif]

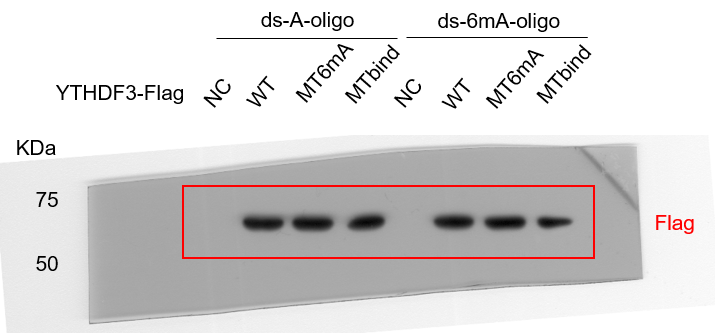

Supplement: Supplementary file 8 — Source data Fig. 5 [file 44318_2025_512_MOESM8_ESM.zip › Figure_5/5F/Flag-Total proteins.tif]

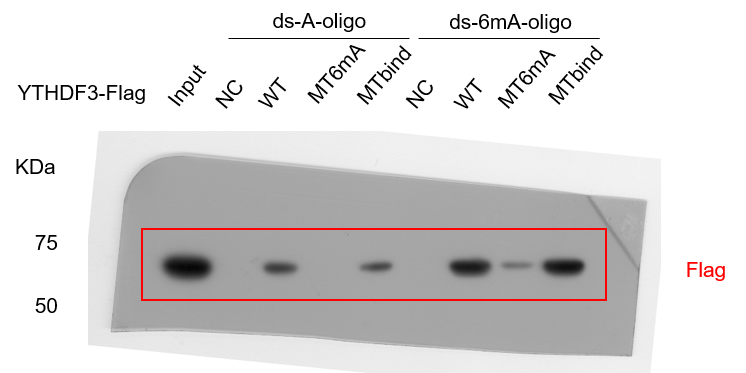

Supplement: Supplementary file 8 — Source data Fig. 5 [file 44318_2025_512_MOESM8_ESM.zip › Figure_5/5F/Flag.tif]

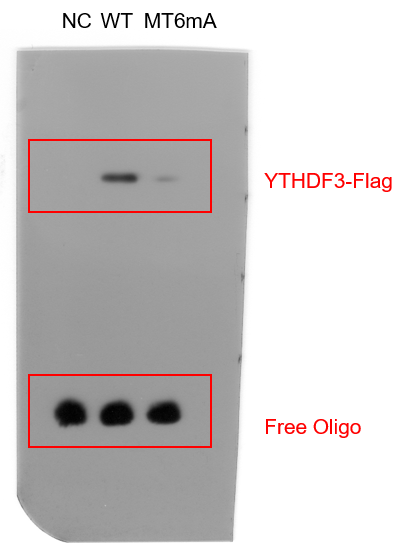

Supplement: Supplementary file 8 — Source data Fig. 5 [file 44318_2025_512_MOESM8_ESM.zip › Figure_5/5G/EMSA.tif]

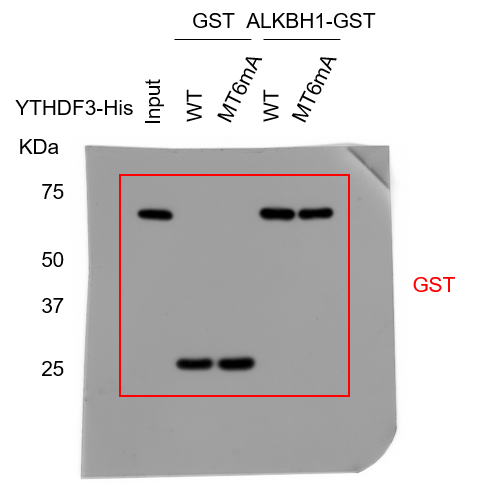

Supplement: Supplementary file 8 — Source data Fig. 5 [file 44318_2025_512_MOESM8_ESM.zip › Figure_5/5H/GST.tif]

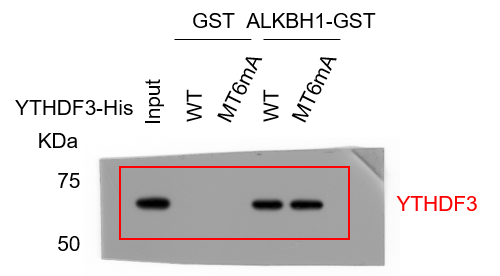

Supplement: Supplementary file 8 — Source data Fig. 5 [file 44318_2025_512_MOESM8_ESM.zip › Figure_5/5H/YTHDF3.tif]

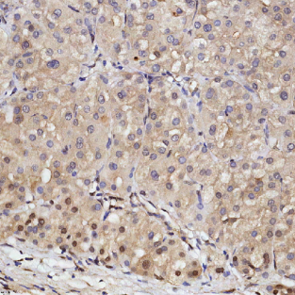

Supplement: Supplementary file 8 — Source data Fig. 5 [file 44318_2025_512_MOESM8_ESM.zip › Figure_5/5I/IHC.tif]

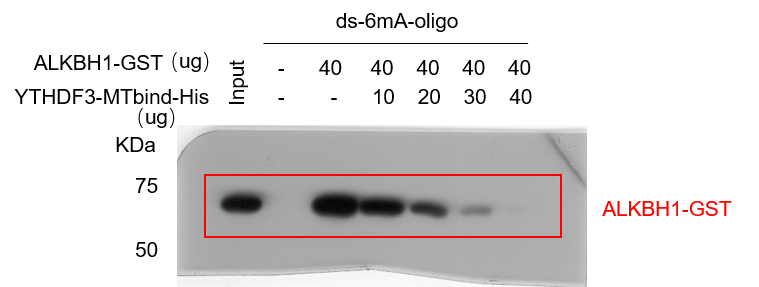

Supplement: Supplementary file 9 — Source data Fig. 7 [file 44318_2025_512_MOESM9_ESM.zip › Figure_7/7A/ALKBH1 (F3-MTbind).tif]

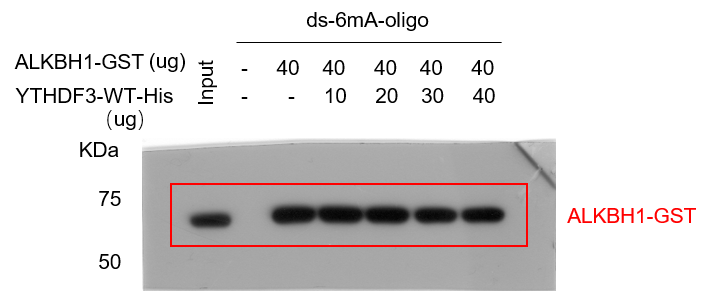

Supplement: Supplementary file 9 — Source data Fig. 7 [file 44318_2025_512_MOESM9_ESM.zip › Figure_7/7A/ALKBH1 (F3-WT).tif]

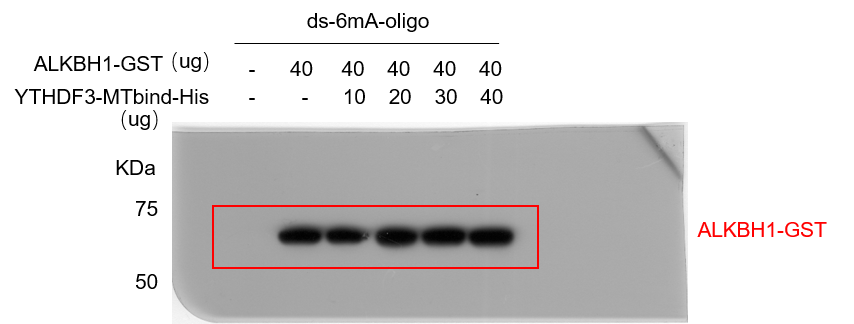

Supplement: Supplementary file 9 — Source data Fig. 7 [file 44318_2025_512_MOESM9_ESM.zip › Figure_7/7A/ALKBH1 - Input(F3-MTbind).tif]

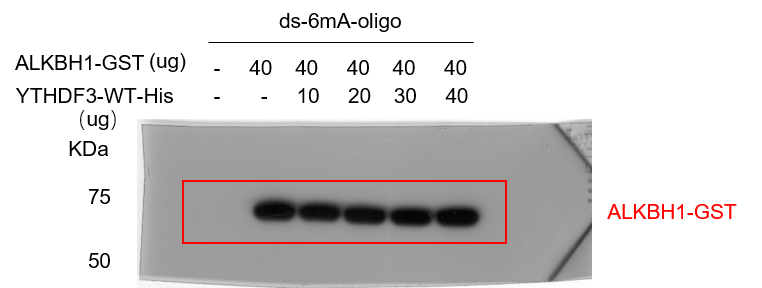

Supplement: Supplementary file 9 — Source data Fig. 7 [file 44318_2025_512_MOESM9_ESM.zip › Figure_7/7A/ALKBH1-Input (F3-WT).tif]

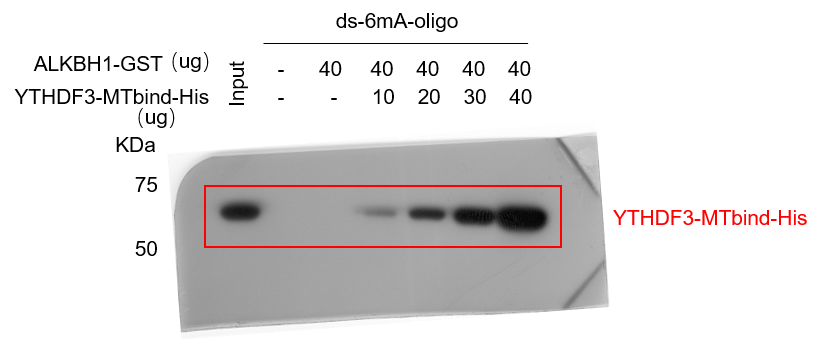

Supplement: Supplementary file 9 — Source data Fig. 7 [file 44318_2025_512_MOESM9_ESM.zip › Figure_7/7A/YTHDF3 (F3-MTbind).tif]

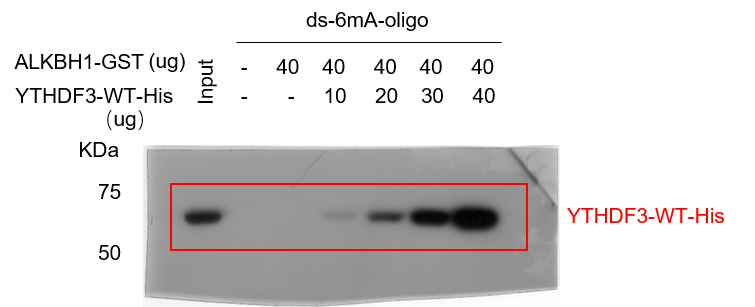

Supplement: Supplementary file 9 — Source data Fig. 7 [file 44318_2025_512_MOESM9_ESM.zip › Figure_7/7A/YTHDF3 (F3-WT).tif]

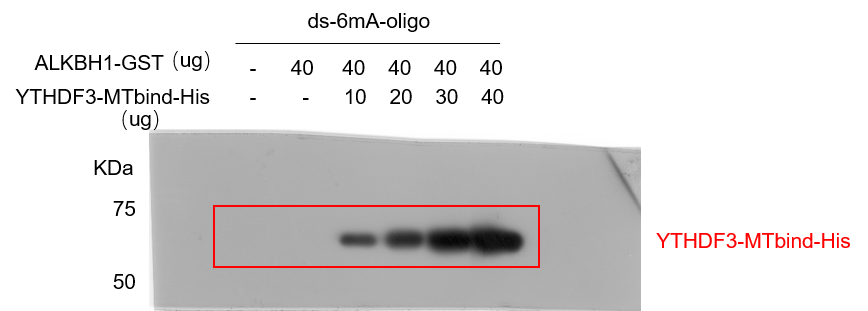

Supplement: Supplementary file 9 — Source data Fig. 7 [file 44318_2025_512_MOESM9_ESM.zip › Figure_7/7A/YTHDF3 - Input (F3-MTbind).tif]

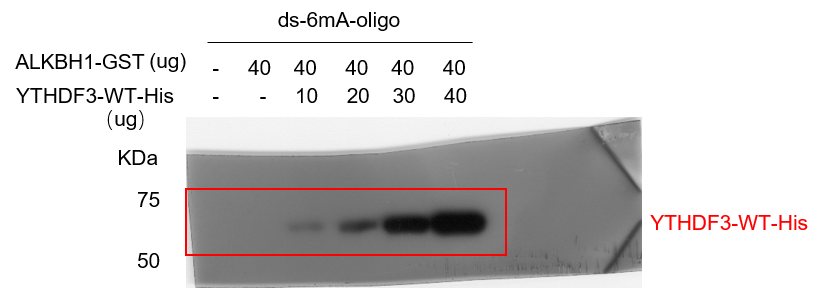

Supplement: Supplementary file 9 — Source data Fig. 7 [file 44318_2025_512_MOESM9_ESM.zip › Figure_7/7A/YTHDF3-Input (F3-WT).tif]

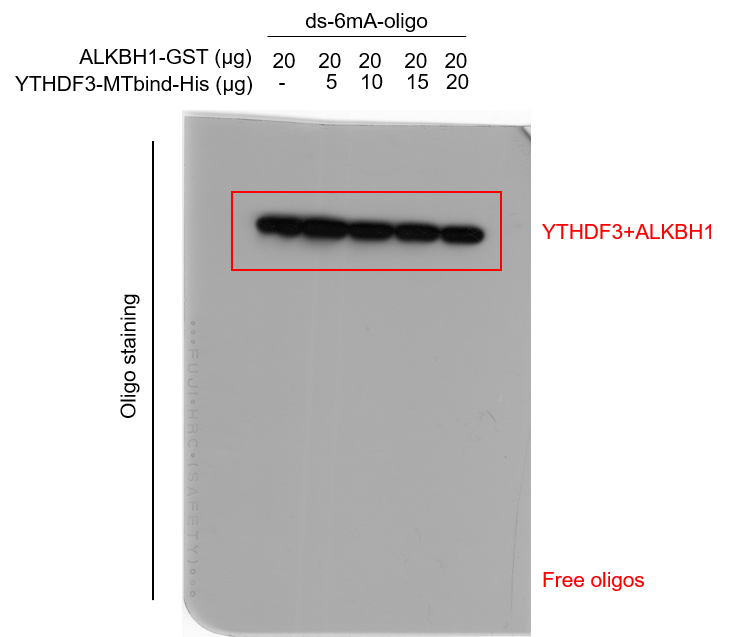

Supplement: Supplementary file 9 — Source data Fig. 7 [file 44318_2025_512_MOESM9_ESM.zip › Figure_7/7B/oligo staining (F3-MTbind).tif]

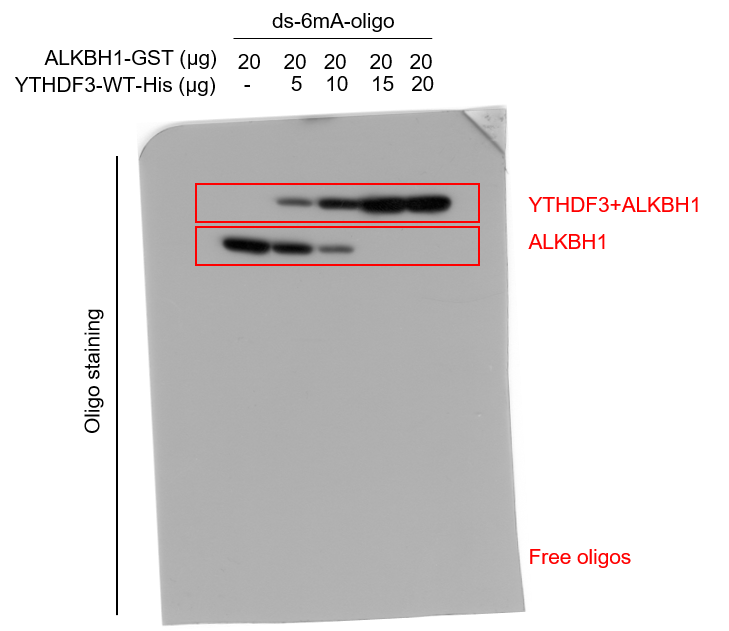

Supplement: Supplementary file 9 — Source data Fig. 7 [file 44318_2025_512_MOESM9_ESM.zip › Figure_7/7B/oligo staining (F3-WT).tif]

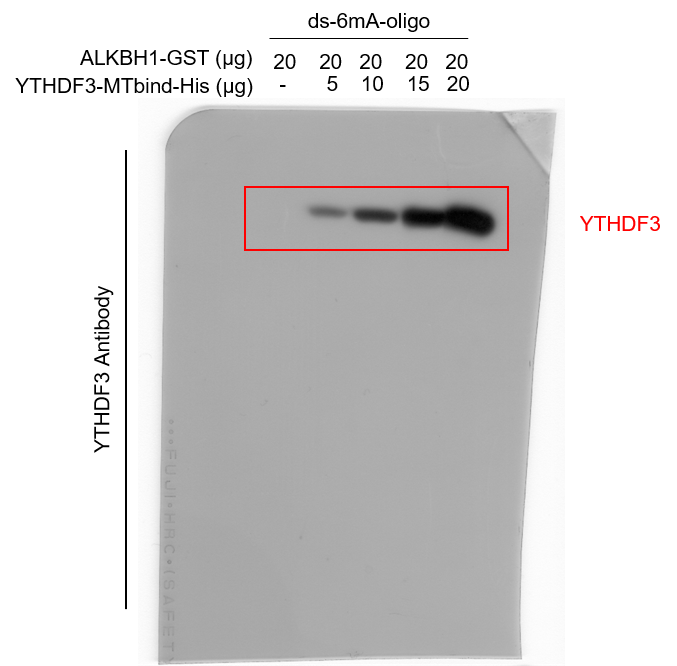

Supplement: Supplementary file 9 — Source data Fig. 7 [file 44318_2025_512_MOESM9_ESM.zip › Figure_7/7B/YTHDF3-Antibody (F3-MTbind).tif]

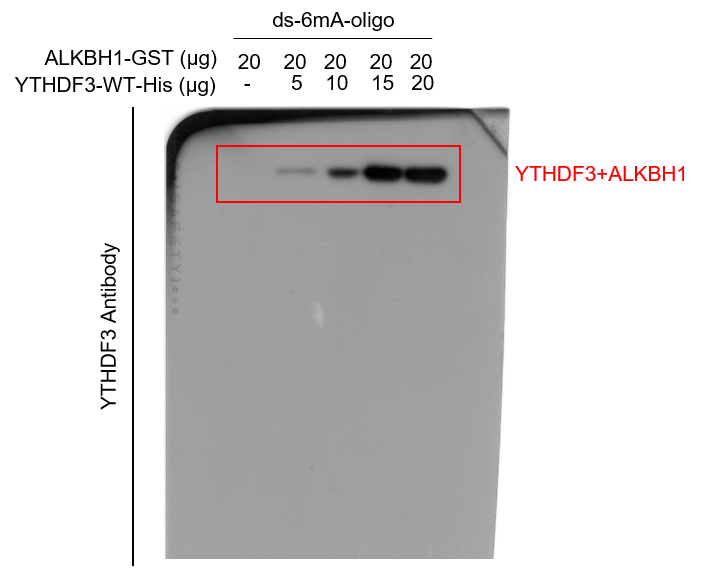

Supplement: Supplementary file 9 — Source data Fig. 7 [file 44318_2025_512_MOESM9_ESM.zip › Figure_7/7B/YTHDF3-Antibody (F3-WT).tif]
