# Supplementary figures and images for: YTHDF3 recognizes DNA N6-methyladenine and recruits ALKBH1 for 6mA removal from genomic DNA (part 2 of 2)
Source: EMBO J. 2025 Jul 25;44(17):4899–917. doi: 10.1038/s44318-025-00512-2 (PMC12402098; doi:10.1038/s44318-025-00512-2)

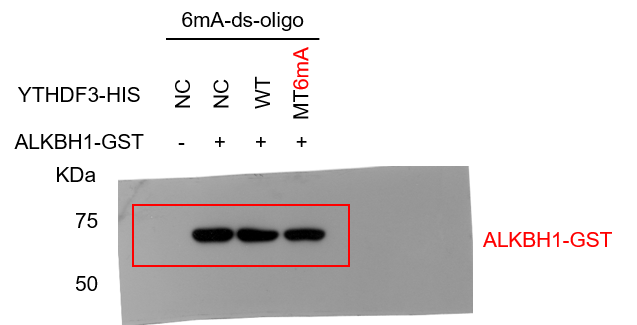

Supplement: Supplementary file 9 — Source data Fig. 7 [file 44318_2025_512_MOESM9_ESM.zip › Figure_7/7C/ALKBH1-GST (Total proteins).tif]

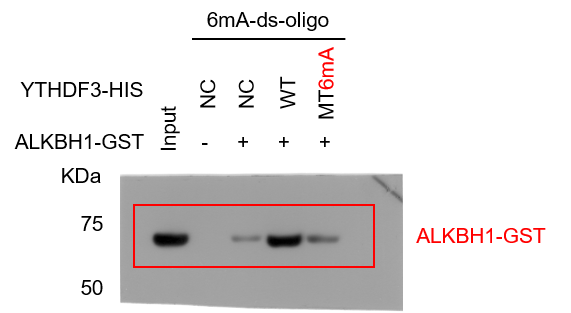

Supplement: Supplementary file 9 — Source data Fig. 7 [file 44318_2025_512_MOESM9_ESM.zip › Figure_7/7C/ALKBH1-GST.tif]

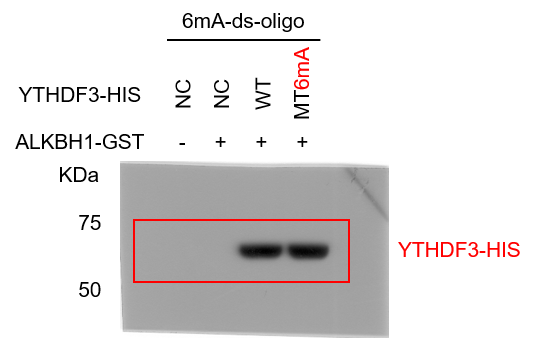

Supplement: Supplementary file 9 — Source data Fig. 7 [file 44318_2025_512_MOESM9_ESM.zip › Figure_7/7C/YTHDF3-His (Total proteins).tif]

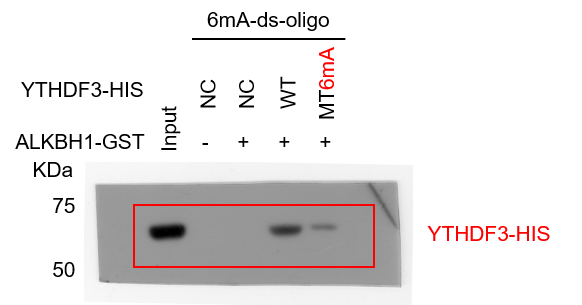

Supplement: Supplementary file 9 — Source data Fig. 7 [file 44318_2025_512_MOESM9_ESM.zip › Figure_7/7C/YTHDF3-His.tif]

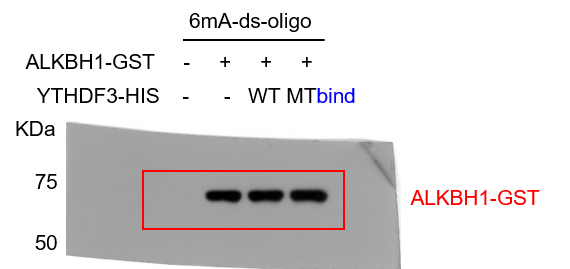

Supplement: Supplementary file 9 — Source data Fig. 7 [file 44318_2025_512_MOESM9_ESM.zip › Figure_7/7E/ALKBH1-GST (Total proteins).tif]

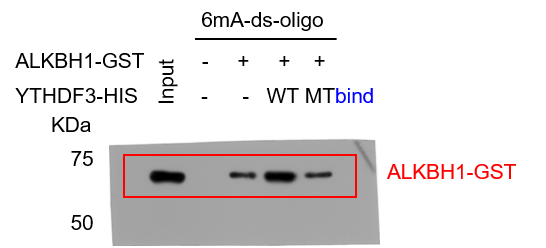

Supplement: Supplementary file 9 — Source data Fig. 7 [file 44318_2025_512_MOESM9_ESM.zip › Figure_7/7E/ALKBH1-GST.tif]

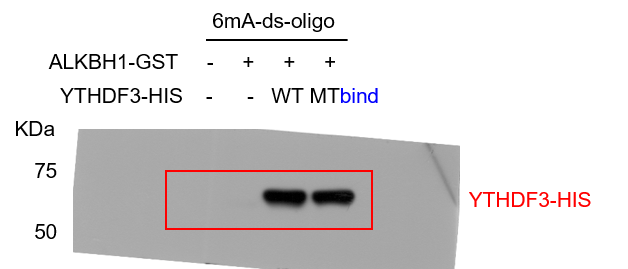

Supplement: Supplementary file 9 — Source data Fig. 7 [file 44318_2025_512_MOESM9_ESM.zip › Figure_7/7E/YTHDF3-HIS (Total proteins).tif]

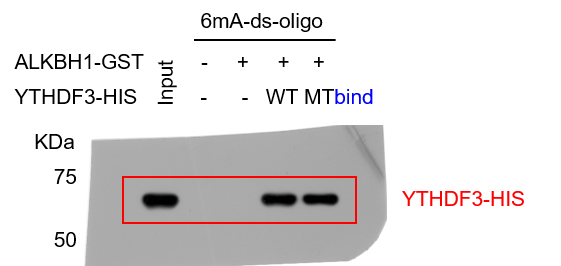

Supplement: Supplementary file 9 — Source data Fig. 7 [file 44318_2025_512_MOESM9_ESM.zip › Figure_7/7E/YTHDF3-HIS.tif]
